# Anti-Inflammatory, Antioxidant Activities, and Phytochemical Characterization of Edible Plants Exerting Synergistic Effects in Human Gastric Epithelial Cells

**DOI:** 10.3390/antiox12030591

**Published:** 2023-02-27

**Authors:** Achille Parfait Nwakiban Atchan, Orissa Charlène Monthe, Armelle Deutou Tchamgoue, Yeshvanthi Singh, Shilpa Talkad Shivashankara, Moorthy Karthika Selvi, Gabriel Agbor Agbor, Paolo Magni, Stefano Piazza, Uma Venkateswaran Manjappara, Jules-Roger Kuiate, Mario Dell’Agli

**Affiliations:** 1 Department of Biochemistry, Faculty of Science, University of Dschang, Dschang P.O. Box 96, Cameroon; 2 Department of Biochemistry, CSIR-Central Food Technological Research Institute (CFTRI), Mysore 570-020, India; 3 Department of Pharmacological and Biomolecular Sciences “Rodolfo Paoletti”, Università degli Studi di Milano, 20133 Milan, Italy; 4 Department of Food Sciences and Nutrition, ENSAI, University of Ngaoundere, Ngaoundere P.O. Box 455, Cameroon; 5 Institute of Medical Research and Medicinal Plants Studies (IMPM), Yaoundé P.O. Box 13033, Cameroon; 6 Department of Microbiology and Immunology, University of Louisville, Louisville, KY 40202, USA; 7 IRCCS MultiMedica, Sesto San Giovanni, 20099 Milan, Italy

**Keywords:** dietary plants, gastric inflammation, interleukin, antioxidant, phenolic compounds, synergistic effect

## Abstract

Dietary bioactive compounds from natural sources (e.g., herbal medicines, foods) are known to potentially suppress acute or chronic inflammation and promote the effectiveness of treatment to reduce the harmful effects of gastritis alone or in combination. In this regard, we have characterized four Cameroonian spice extracts, namely *Aframomum citratum*, *Dichrostachys glomerata*, *Tetrapleura tetraptera*, and *Xylopia parviflora* through reverse phase-high-performance liquid chromatography (RP-HPLC), ultra-performance liquid chromatography-electrospray ionization high-resolution mass spectrometry (UPLC-ESI-HRMS/MS), and Fourier transform infrared spectroscopic (FTIR) analyses and investigated their antioxidant and synergistic anti-inflammatory activities in human gastric adenocarcinoma (AGS) and gastric epithelial (GES-1) cells. The extracts showed a high amount of total phenolic (TPC: 150–290 mg gallic acid equivalents (GAE)/g of extract) and flavonoid content (TFC: 35–115 mg catechin equivalents (CE)/g of extract) with antioxidant properties in a cell-free system (1,1-Diphenyl-2-picryl-hydrazyl (DPPH) half maximal inhibitory concentration (IC_50_s) ≤ 45 µg/mL; 2,2′-azinobis-(3-ethylbenzothiazoline-6-sulfonic acid) (ABTS) half maximal inhibitory concentration (IC_50_s) ≤ 29 µg/mL. The extracts in combination (MIX) exert a synergistic beneficial effect (combination index (CIs) < 1 and dose reduction index (DRIs) > 1) on inflammatory markers (interleukin (IL)-8 and -6 release, and nuclear factor kappa B (NF-κB) driven transcription) in human gastric epithelial cells, which may result from the presence of phenolic compounds (phenolic acids, flavonoids) or other compounds (protein, lipid, aromatic, and polysaccharide compounds) tentatively identified in the extracts. The general findings of the present study provide supporting evidence on the chemical composition of four Cameroonian dietary plants and their significant synergistic inhibitory activities on inflammatory markers of gastric epithelial cells.

## 1. Introduction

Gastric ulcers and gastritis are among the most common gastrointestinal disorders, with a strong relationship with *Helicobacter pylori* (*H. pylori*) infection and nonsteroidal anti-inflammatory drugs (NSAID) administration [1]. Various pathogenic mechanisms can contribute to the development of ulcers leading to an imbalance between protective (prostaglandins, mucus, endogenous antioxidants, etc.) and aggressive (hydrogen chloride (HCl), reactive oxygen species, pepsin) factors occurring in the gastric mucosa [2]. Genetic predisposition, smoking, stress, nutritional deficiencies, alcohol consumption, in addition to the long-term use of NSAIDs (i.e., acetylsalicylic acid or indomethacin) and *H. pylori* infection are all causative factors relevant to the development of gastric ulcers [3].

From a mechanistic point of view, several transcription factors are involved in gastric inflammatory disorders, with nuclear factor kappa B (NF-κB) playing a key role among others. Several in vitro studies, also performed by our group, have shown that *H. pylori* and some pro-inflammatory cytokines (i.e., tumor necrosis factor (TNF)-α) are capable of activating the NF-κB pathway in gastric epithelial cells [4,5]. NF-κB is responsible for the expression and release of IL-8 and IL-6, which, in turn, increase the gastric phlogistic processes [4].

The prevention or treatment of gastric ulcers is a medical challenge [6]. Currently, the treatment of gastric ulcers and gastritis requires a combination of drugs, such as proton pump inhibitors (i.e., omeprazole), histamine receptor antagonists (i.e., ranitidine), anticholinergic, and antibiotics (i.e., clarithromycin, amoxicillin, and metronidazole). Although it is possible to achieve effectiveness with the clinical use of these drugs, their potential side effects and drug interactions are major problems during treatment, thus making drug tolerance by patients quite low [7]. Consequently, products with good efficacy and negligible or no side effects are necessary. Several studies have shown that natural products from herbal medications have therapeutic benefits for gastric disorders with fewer side effects [8,9,10]. Moreover, the use of plant extracts in combination (synergistic therapy) may lead to new therapeutical strategies and represent a potential area for future investigations [11].

Since ancient times, nature has supplied a variety of phytochemicals with beneficial effects on humans. Thus, there is increasing attention to natural products, especially for the treatment of gastrointestinal disorders, corroborated by the traditional use, low cost, and lower toxicity with respect to conventional medicines [12]. Based on ethnopharmacological information, and our previous studies, in which we reported the gastro-protective and anti-inflammatory effects of a variety of Cameroonian plants in human epithelial cells (GES-1 and AGS) [5,13], *Xylopia parviflora Spruce*, *Tetrapleura tetraptera* (Schum. and Thonn.) Taub, *Dichrostachys glomerata* (Forssk.) Chiov., and *Aframomum citratum* (C.Pereira) K.Schum were chosen for gastro-protective evaluation. Our early studies showed that hydro-ethanolic extracts from these plants exerted antioxidant, hepato-protective, and enzyme inhibition activities [14,15,16,17]. In addition to their anti-inflammatory effects, the combination of these dietary plants, suggested by traditional Cameroonian medicine, might provide phytochemicals (mainly polyphenols) with synergistic gastro-protective effects. In this report, we chemically characterized the compounds present in each extract through the reversed-phase-high performance liquid chromatography (RP-HPLC), ultra-performance liquid chromatography-triple time-of-flight electrospray ionization tandem mass spectroscopy (UPLC-Triple TOF-ESI-MS/MS), and Fourier transform infrared spectrophotometer (FTIR) analyses. In addition, the gastro-protective activity and mode of action of the extracts in combination were investigated in human GES-1 and AGS cells.

## 2. Materials and Methods

### 2.1. Chemicals and Reagents

The HPLC-grade solvents were purchased from Spectrochem Pvt. Ltd., Mumbai (India). MilliQ water prepared by a Millipore water purification system (Merck, Mumbai, India) and ultrapure double distilled were used to prepare reagents and buffers throughout the experiment to prevent metal contamination. HPLC analytical grade syringic acid, chlorogenic acid, gallic acid, protocatechuic acid, p-hydroxybenzoic acid, catechin, caffeic acid, coumaric acid, epicatechin, gallate, quercetin, ferulic acid, kaempferol were used for chromatographic analysis. All remaining chemicals and solvents were obtained from Sigma Chemicals Co. (St. Louis, MO, USA). Human epithelial adenocarcinoma cells (AGS, CRL-1739) were purchased from LGC Standard S.r.l. (Milan, Italy). Gastric non-tumoral epithelial cells (GES-1) were a kind gift from Dr. Dawit Kidane-Mulat (University of Texas, Austin, TX, USA). Roswell Park Memorial Institute (RPMI 1640) medium and Dulbecco’s Modified Eagle’s Medium/F12 (DMEM)/F12 (1:1) were purchased from Gibco (Life Technologies Italia, Monza, Italy). 1,1-Diphenyl-2-picryl-hydrazyl (DPPH), 3,3′,5,5′-tetramethylbenzidine (TMB), 2,2′-azinobis-(3-ethylbenzothiazoline-6-sulfonic acid) (ABTS), Folin–Ciocalteu reagent (FCR), and 3-(4,5-dimethylthiazol-2-yl)-2,5 diphenyltetrazolium bromide (MTT) were from Sigma Aldrich (Milan, Italy). Life Technologies Italia (Monza, Italy) provided penicillin, streptomycin, L-glutamine, sodium pyruvate, trypsin-EDTA, and Lipofectamine 2000. Human IL-8 and IL-6 ELISA Development Kits were manufactured by Peprotech Inc. (London, UK). Fetal bovine serum (FBS) and disposable materials for cell culture were purchased by Euroclone (Euroclone S.p.A., Pero-Milan, Italy). All reagents were at the highest grade available.

### 2.2. Sample Preparation

*Xylopia parviflora* Spruce, *Tetrapleura tetraptera* (Schum. and Thonn.) Taub, *Dichrostachys glomerata* (Forssk.) Chiov., and *Aframomum citratum* (C.Pereira) K.Schum were harvested as previously described by Nwakiban et al. [14,18]. The plant material was cleaned, taken to dryness, and stored at room temperature. The air-dried and powdered samples (100 g) were subjected to magnetic stirring with 100 mL of a hydroalcoholic (ethanol: water, 70:30) mixture for 4 and 6 hrs at room temperature, in dark conditions. The solvent was removed through a rotary evaporator (Laborota 4000 efficient, Heidolph Instruments, Schwabach, Germany), and subjected to lyophilization. The lyophilized extracts were maintained at 4 °C, and 10 mg were freshly dissolved in 1 mL of HPLC grade or MS grade methanol for chemical characterization. Each extract and its combination (MIX) (mixed in the same proportion) were dissolved in pure dimethyl sulfoxide (DMSO), aliquoted, and stored at −80 °C. For cell treatment and anti-inflammatory assays under sterile conditions, the final concentration of DMSO added to the cells was not above 0.1%.

### 2.3. Quantification of Phenolic Compounds through HPLC-PDA

The phenolic compounds content within each extract was analyzed as described above [17] by HPLC (Nexera X-2 LC-30A, Shimadzu, Japan), and chromatography separations were carried out on a Chromasol™ RP-C18 column (250 mm × 4.6 mm, 5 µm). The mobile phase was made up of water set at a pH of 2.65 with acetic acid (solvent A) and solvent B (20% solvent A and 80% acetonitrile). The extracts were dissolved in HPLC-grade methanol and filtered through a syringe filter (0.45 µm PVDF, Millipore, MA, USA). A 20 µL volume for each standard or sample was injected into the HPLC system, and a linear elution gradient was applied in the following manner: 0–20% B in 0–35 min, 20–50% B in 35–40 min, 50–100% B in 40–45 min, and 100–0% B in 45–60 min. The temperature of the column was kept at 20 °C. The flow rate was 1.2 mL/min, and the PDA detector was adjusted to λ_1_ = 280 and λ_2_ = 320 simultaneously using LabSolutions software (version 6.50, Shimadzu, Japan). The identified phenolic compounds were quantified using the peak area obtained from the standards, comparing their retention times with those of corresponding standards and by spiking samples with appropriate standards.

### 2.4. UPLC-ESI-HRMS/MS Analysis

The extracts were diluted with MS-grade methanol and injected directly into the UPLC-ESI-HRMS/MS system with an ekspert 110 binary pump, an ekspert 110-XL autosampler, an ekspert PDA detector, and ekspert 110 column compartment (AB Sciex Instruments, Netherlands) via a syringe pump. The Kinetex C18 100A (30 × 2.1 mm 1.7 μm) Column (Phenomenex) was used for separation at a flow rate of 0.4 mL/min. The mobile phase was made up of a combination of two solvents: A (0.1% acetic acid in water) and B (0.1% acetic acid in acetonitrile and methanol in the ratio 8:2) [17]. The mass spectra analyses were performed in negative ion mode in the *m/z* range from 100 to 2000 *m/z* at an IRDx resolution of 15,000 using an LC/MS/MS Quadrupole-TOF Hybrid Mass Spectrometer (Sciex Triple ToF 5600, Singapore) and under the following conditions: gases were GS1-45, GS2-60, and Curtain GAS (CUR)-40, source voltage was 4.0 kV, Duospray ion source was set at 400 °C, and the collision energy was 10 V. CID-MS/MS experiments were carried out on mass-selected precursor ions using standard isolation and excitation configuration. All data acquisition and analysis were performed using the Peak View 2.1 Software (AB SCIEX Triple TOF 5600, Singapore), which has MasterView™ (Version 1.0, AB SCIEX). The Master View’s XIC management tool was used to detect quasi-molecular weights, mass errors, and isotope patterns of both targeted and non-targeted compounds. ChemSpider, elemental composition analysis, as well as literature review, were used to define consistent tentative structures of the identified compounds.

### 2.5. Fourier Transform Infrared Spectroscopic (FTIR) Analysis

Dried powder from various extracts of each plant material was used for FTIR analysis. Approximately 10 mg of each extract were loaded into the FTIR spectroscope (Bruker, Germany), which is connected to a computer operating on a Windows system and to an OPUS software (Version 7.0 Bruker Optic). The spectra were recorded at a resolution of 4 cm^−1^ from 4000 to 400 cm^−1^ using 64 co-added scans. All spectra were subtracted against a background of air spectra. The attenuated total reflectance (ATR) plate was carefully cleaned by scrubbing with isopropyl 70% twice, then drying with soft tissue before being filled with the following sample. The spectra were recorded as absorbance values at each data point in triplicate and two times for spectrum confirmation.

### 2.6. Estimation of Polyphenols and Antioxidant Activity Assays

The polyphenol estimation and antioxidant capacity assays were modified and translated into 96-well plates based on the methods used in previous literature reports [19,20,21]. The stock solution (100 μg/mL) of each extract was prepared for experimental analysis and the data was measured using a UV–VIS spectrophotometer (Shimadzu, Model: UV 2100, Kyoto, Japan). All tests were run in triplicate at least three times.

#### 2.6.1. Total Phenolic Content Assay

Total phenolic content (TPC) was determined using a Folin-Ciocalteu assay with slight modifications [22]. Briefly, a 25 µL sample was mixed with 25 µL Folin’s reagent (1:3 diluted with water), and 200 µL deionized water was added, then the mixture was incubated at room temperature for 5 min. The reaction mixture was then alkalized by adding 25 µL of sodium carbonate (10%, *w/v*) and allowed to stand in the dark at 25 °C for 60 min. The absorbance was read at 765 nm against the reagent blank. Gallic acid was used as a standard in a concentration range between 0 and 50 µg/L. TPC was expressed in mg gallic acid equivalents (GAE) per g of dried extracts based on the calibration curve.

#### 2.6.2. Total Flavonoid Content Assay

Total flavonoid content (TFC) was determined by the colorimetric method as described by Zhishen et al. [23] and slightly modified by Moukette et al. [24]. To a 15 µL sample or standard, 45 µL of distilled water, followed by 4.5 µL of sodium acetate (5% solution) were added. The mixture was left in the darkness at 25 °C for 5 min and 4.5 µL of aluminum chloride (10% of Al_2_Cl_3_) was added. Afterward, 30 µL of 1 mM NaOH and 150 µL distilled water were added to the reaction mixture. The absorbance of the resulting solution was measured at 765 nm wavelength against the reagent blank. Catechin was used as a standard in a concentration range between 0 and 60 µg/L. TFC was expressed in mg catechin equivalents (CE) per g of dried extracts based on the calibration curve.

#### 2.6.3. Total Flavonol Content Assay

The total flavonol assay (FC) was adapted from [25,26] with quercetin as the standard compound. Briefly, 80 µL of the samples were mixed with 80 µL of aluminum chloride (2% of Al_2_Cl_3_) diluted in ethanol and 120 µL of 50 g/L sodium acetate solution. The mixture was incubated at 25 °C for 2.5 hrs and the absorbance was measured at 440 nm. Concentrations (0–60 µg/mL) of quercetin dissolved in ethanol were used to draw the standard curve. The results were expressed as mg quercetin equivalents (QE) per g of dried extracts based on the calibration curve.

#### 2.6.4. 1,1-Diphenyl-2-picryl-hydrazyl (DPPH) Assay

The DPPH scavenging activity of extracts was determined based on the method of Sogi et al. [27] and modified from the method reported by Peng et al. [20]. Samples were dissolved in methanol and tested at concentrations between 1 and 100 µg/mL. An aliquot of a 40 µL sample was mixed with 260 µL of 0.1 mM DPPH radical methanolic solution in a 96-well plate and incubated for 30 min at 25 °C. Afterward, the absorbance was measured at 517 nm against the reagent blank. Concentrations ranging from 0 to 50 µg/mL ascorbic acid dissolved in water were used to draw the standard curve. The results were expressed as mg ascorbic acid equivalents (AAE) per g of dried extracts and the IC_50_ concentration showing 50% radical scavenging activity was determined.

#### 2.6.5. 2,2′-Azinobis-(3-ethylbenzothiazoline-6-sulfonic Acid) (ABTS) Assay

The ABTS antioxidant activity of spice extracts was carried out using the ABTS^+^ radical cation decolorization assay with some modifications [28]. To 5 mL of 7 mmol/L of ABTS solution, 88 µL of a 140 mM potassium persulfate solution was added to produce ABTS^+^. The mixture was allowed to stand in the dark at 25 °C for 16 hrs, then 0.5 mL of the ABTS^+^ solution was diluted by adding 45 mL analytical grade ethanol to obtain an initial absorbance of 0.70 ± 0.02 at 734 nm. A 10 µL sample extract and 290 µL prepared ABTS^+^ solution were mixed in a 96-well plate and incubated at 25 °C for 6 min in the darkness. The absorbance was measured at 734 nm against the reagent blank. Concentrations from 0 to 15 µg/mL Trolox were used to draw the standard curve. The results were expressed as mg Trolox equivalents (TE) per g of dried extracts and the IC_50_ concentration showing 50% radical scavenging activity was determined.

### 2.7. Cell Culture

Human non-tumoral gastric epithelial cells (GES-1) and human adenocarcinoma gastric epithelial cells (AGS) were respectively grown in RPMI 1640 and DMEM/F12 media supplemented with L-glutamine 2 mM, streptomycin 100 mg/mL, penicillin 100 units/mL, and 10% heat-inactivated fetal bovine serum, at 37 °C in a humidified atmosphere containing 5% CO_2_.

### 2.8. Cytotoxicity Assay and Cell Treatment

Cell viability was measured as previously described by Nwakiban et al. [5], after 6 h of co-treatment with the stimulus (TNFα, 10 ng/mL) and the combination (MIX) of extracts (assessed in the range 0.1–10 μg/mL), by the 3,4,5-dimethylthiazol-2-yl-2,5-diphenyltetrazolium bromide (MTT) method. Briefly, the medium was removed from each well at the end of the treatment and 200 μL of MTT solution (0.1 mg/mL) was added for 45 min at 37 °C in dark conditions. The formazan salt was extracted from the cells with 200 μL of a mixture of DMSO: isopropanol (10:90), and the absorbance was measured at 570 nm (Envision, PerkinElmer, Walthman, MA, 02451, USA). To study the release of pro-inflammatory mediators (IL-8 and IL-6) and the NF-κB driven transcription, cells were seeded in 24-well plates at a density of 30,000 cells/well. Seventy-two hours later, cells were co-treated with the pro-inflammatory stimulus (TNFα, 10 ng/mL) and the MIX extracts for 6 h using serum-free medium: DMEM/F12 or RPMI 1640 medium, supplemented with penicillin 100 units/mL, L-glutamine 2 mM, and streptomycin 100 mg/mL. At the end of the treatment, the media were collected for the biological assays. All tests were run in triplicate at least three times.

### 2.9. Transient Transfection Assay

Gastric epithelial cells were transiently transfected with the reporter plasmid NF-κB-Luc [29], which contains three κB responsive elements controlling the luciferase gene. AGS cells were transfected by the calcium phosphate method, whereas GES-1 cells were transfected using Lipofectamine^®^ (Invitrogen, Thermo Fisher Scientific, Waltham, MA, USA). The day after, the cells were treated for 6 hrs with the MIX extracts and TNFα (10 ng/mL), as previously described. Then, cell lysis and the luciferase enzymatic reaction were conducted through Britelite^TM^ Plus reagent (PerkinElmer Inc., Milan, Italy), according to the manufacturer’s instructions. The results (mean ± SD of at least three experiments) were expressed as relative% with respect to the luminescence of the pro-inflammatory conditions (100%).

### 2.10. Measurement of IL-8 and IL-6 Secretion

IL-8 and IL-6 were quantified using two different ELISA kits, as previously reported [5], according to the manufacturer’s instructions. Briefly, Corning 96 well EIA/RIA plates (Sigma-Aldrich, St. Louis, MO, USA) were coated with the corresponding antibody and incubated overnight at room temperature. Then, cell media and biotinylated antibodies were added to construct a sandwich ELISA. The cytokines were measured in the samples at 450 nm through the colorimetric reaction between horseradish peroxidase enzyme and 3,3′,5,5′-tetramethylbenzidine substrate (Sigma-Aldrich, St. Louis, MO, USA) using a spectrophotometer (Victor X3, PerkinElmer, Walthman, MA, 02451, USA). Epigallocatechin-3-O-gallate (EGCG, 20 μM) was used as a reference inhibitor, for its ability to decrease both TNFα-induced IL-8 and IL-6 secretion [4,5,9].

### 2.11. Synergistic Effect Analysis of Extracts in Combination

The Chou Talalay equations [30,31] and the CompuSyn software (version 1.0; ComboSyn, Paramus, NJ, USA) were used to determine the combination index (CI) and the dose reduction index (DRI). The half-maximal inhibitory concentration (IC_50_) (µg/mL) of every single extract on IL-6, IL-8, and NF-κB has already been obtained during our latest research work [5]. The CI was used to determine the types of drug interactions in which CI < 1 indicates a synergistic effect, CI = 1 indicates an additive effect, and CI > 1 represents an antagonistic effect. The Equation (1) below was used to calculate the CI for the combination of the extracts:

(1) $$\mathrm{CI}=\frac{{\mathrm{IC}}_{50}\;\mathrm{of}\;\mathrm{Extract}\;A\;\mathrm{in}\;\mathrm{combination}}{{\mathrm{IC}}_{50}\;\mathrm{of}\;\mathrm{Extract}\;A\;\mathrm{in}\;\mathrm{monotherapy}}+\ldots +\frac{{\mathrm{IC}}_{50}\;\mathrm{of}\;\mathrm{Extract}\;D\;\mathrm{in}\;\mathrm{combination}}{{\mathrm{IC}}_{50}\;\mathrm{of}\;\mathrm{Extract}\;D\;\mathrm{in}\;\mathrm{monotherapy}}$$

The dose reduction index (DRI) was calculated using the following Formula (2), by measuring how many times each extract could be reduced in the combination compared to monotherapy:

(2) $${\mathrm{DRI}}_{\mathrm{Extract}\;A}=\frac{{\mathrm{IC}}_{50}\;\mathrm{of}\;\mathrm{Extract}\;A\;\mathrm{in}\;\mathrm{monotherapy}}{{\mathrm{IC}}_{50}\;\mathrm{of}\;\mathrm{Extract}\;A\;\mathrm{in}\;\mathrm{combination}}$$

### 2.12. Statistical Analysis

All results are expressed as mean ± SD. Statistical data were determined with a one-way analysis of variance (ANOVA) followed by the multiple comparison analysis performed with the Bonferroni post hoc test. To compare data obtained from RP-HPLC, the Waller–Duncan test of SPSS software (IBM Corporation, NY, USA, Version 25) was used to test for differences in means. Pearson’s test was applied to understand the correlation between the antioxidant variables through the XLSAT software (Version 2022). Statistical analyses were calculated, and graphs were prepared using GraphPad Prism 9.0 software (GraphPad Software Inc., San Diego, CA, USA).

## 3. Results and Discussion

### 3.1. Characterization of Compounds in Extracts by UPLC-ESI-HRMS/MS Analysis

The UPLC-ESI-HRMS/MS profiles of *X. parviflora*, *D. glomerata*, *T. tetraptera,* and *A. citratum* hydro-ethanolic extracts are shown in Figure S1 and the chromatographic, MS, and MS/MS data are reported in Table 1. The structure of 33 compounds present in the four spice extracts from Cameroon was tentatively identified using the combined interpretation of the fragmentation patterns and the retention time obtained from the UPLC-ESI-HRMS/MS analysis.

#### 3.1.1. Phenolic Acids

Nine compounds belonging to two different classes of phenolic acids have been identified in the extracts (two hydroxybenzoic acids and six hydroxycinnamic acids) (Table 1). Caftaric acid derivatives were identified in *D. glomerata* (compound 9b, t_R_ = 16.05 min) and *T. tetraptera* (compound 11c, t_R_ = 16.03 min) extracts with [M−H]^−^ at *m/z* 311.2 (Figure S1B,C). They displayed similar MS^2^ fragmentation patterns (Table 1) that produced a fraction at *m/z* 149 [M−H−162]^−^, thus indicating the loss of a caffeoyl moiety [32]. Protocatechuic acid 4-O-glucoside (compound 4a, t_R_ = 15.25 min), gallic acid monohydrate (compound 1a, t_R_ = 11.59 min), and derivative (compound 7a, t_R_ = 17.53 min) were identified as the hydroxybenzoic acids present in *A. citratum* (Figure S1C). Compound 1a showed a [M−H]^−^ at *m/z* 187.1 and it produced two fractions, one base peak at *m/z* 125 [M−H−CO_2_−H_2_O]^−^ and another at *m/z* 169 [M−H−H_2_O]^−^, characteristics of gallic acid [33]. Compound 7a, with a [M−H]^−^ at *m/z* 473.3 and fragment at *m/z* 311 [M−H−162]^−^, obtained after a loss of caffeoyl moiety, was assigned as chicoric acid, a hydroxycinnamic acid derivative, while compound 4a displayed parent ion at *m/z* 315.3 and fragment ion at *m/z* 141 [M−H−162−CO]^−^ with the cleavage of the O-sugar bond (Table 1) [21,34]. Compound 8b (t_R_ = 15.10 min) and compound 5a (t_R_ = 15.75 min) (Figure S1B,C) revealed molecular ions at *m/z* 325.2 and *m/z* 295.2, respectively (Table 1). Based on these data and comparing retention times and MS^2^ fragments, they were tentatively assigned to hydroxycinnamic acid compounds [17,21]. Furthermore, compound 6d (t_R_ = 12.58 min) and compound 10d (t_R_ = 15.65 min) (Figure S1D) in *X. parviflora* spectra exhibited [M−H]^−^ at *m/z* 335.2 and *m/z* 339.2, respectively (Table 1) The MS^2^ fragmentation patterns of compound 6d are typical of caffeoylshikimic acid [35]. Compound 10d showed fragment ions at *m/z* 183 [M−H−156]^−^ characterized for methylgallate residue, and at *m/z* 197 [M−H−146]^−^ corresponding to the loss of one deoxyhexose, which indicated the presence of caffeoyl tricarboxylic acid isomers [33]. Compound 11d (t_R_ = 16.96 min) was conditionally identified as an isomer of ellagic acid with a deprotonated molecule at *m/z* 301.2, based on its fragmentation pattern, including the characteristic aglycone fragment (Figure S1D) [36].

#### 3.1.2. Flavonoids

Flavonoids were present mostly as anthocyanins, flavanols, flavanonols, flavanones, flavones, flavonols, and isoflavonoids (Table 1).

Ten flavanols (compounds 5c, 7c, 8c, 1c, 2c, 3c, 4c, 2d, 8d and 9d) were identified in the hydro-ethanolic extracts. Compound 5c ([M−H]^−^ at *m/z* 841.5, t_R_ = 12.54 min), compound 7c ([M−H]^−^ at *m/z* 865.5, t_R_ = 13.49 min), and compound 8c ([M−H]^−^ at *m/z* 825.5, t_R_ = 13.60 min) were tentatively assigned as B-type proanthocyanidin trimers (Figure S1C), due to the fragmentation sequences of their molecular ions which yielded MS^2^ ions at 441 [M−H−162−162−76]^−^, 751 [M−H−90]^−^ for compound 5c, 658 [M−H−162−CH_2_O−CH_3_]^−^, 640 [M−H−120−90−CH_3_]^−^ for compounds 7c and 617 [M−H−120−2CO_2_]^−^, 735 [M−H−90]^−^ for compounds 8c, indicating the presence of a C-hexosyl unit that producing 0, 2 and 0, 3 cross ring cleavage (Table 1) [37,38]. Compound 1b (t_R_ = 8.36 min) and compound 2d (t_R_ = 8.41 min) (Figure S1B,D), with similar [M−H]^−^ at *m/z* 289.1, showed characteristic MS^2^ fragments at *m/z* 245 [M−H−CO_2_]^−^ (loss of carboxyl group), *m/z* 109 [M−H−162−H_2_O]^−^ (loss of a caffeoyl moiety and water), and *m/z* 203 [M−H−60− CO_2_]^−^ (cleavage of the A-ring of flavan-3-ol) (Table 1). Therefore, those compounds were identified as (+)-catechin [33,38]. Another compound was detected at t_R_ = 8.94 min (compound 2b) in *D. glomerata* (Figure S1B) and exhibited a [M−H]^−^ at *m/z* 561.2. The MS^2^ spectrum gave intense ions at *m/z* 271 [M−H−288]^−^ (loss of galloyl residue), and other fragment ions at *m/z* 289 and *m/z* 245, which are similar to those obtained for compound 1b (Table 1). These results led to identifying compound 2b as a catechin monogallate [38]. Compound 3b (t_R_ = 9.14 min) with a [M−H]^−^ at *m/z* 729.2 generated an MS^2^ fragment ion at *m/z* 289 [M−H−152−288]− corresponding to the loss of a galloyl group followed by the loss of an (epi)catechin molecule or to the loss of an (epi)catechin gallate (*m/z* 289 [M−H−152−288]^−^) (Table 1). Based on the obtained mass spectrometry sequences, this compound was tentatively identified as procyanidin dimer monogallate [38,39]. The MS^2^ spectrum of compound 4b (t_R_ = 9.62 min), with [M−H]^−^ at *m/z* 441.2 produced ions at *m/z* 169 [M−H−152−120] and 289 [M−H−152]^−^ (Table 1) corresponding to the deprotonated ions of gallic acid and (epi)catechin, respectively. Compound 4b also displayed similar MS^2^ fragmentation patterns that produced a fraction at *m/z* 245 and *m/z* 271 which is characteristic of (epi)catechin monogallate [19]. Compound 8d (t_R_ = 14.37 min) (Figure S1A), with [M−H]^−^ at *m/z* 315.2 was identified as an isomer of isorhamnetin by comparison of their MS^2^ fragmentation spectra [33]. Compound 9d (t_R_ = 15.33 min) showed a [M−H]^−^ at 505.2 and produced fragmentation sequences at *m/z* 359 [M−H−146]^−^, *m/z* 373 [M−H−132]^−^, *m/z* 417 [M−H−2CO_2_]^−^ corresponding to the loss of a pentoxyl and deoxyhexosyl unit (Table 1). Based on these data, it was assigned to quercetin 7-O-pentosyl-deoxyglucoside.

Four flavanones, compounds 1c (t_R_ = 11.56 min), compounds 5b (t_R_ = 10.32 min), 2a (t_R_ = 11.72 min), and 4d (t_R_ = 10.14 min) were respectively identified in *T. tetraptera*, *D. glomerata*, *A. citratum,* and *X. parviflora* extracts (Figure S1A–D). Compounds 5b, 2a, and 4d showed similar [M−H]^−^ at *m/z* 433.1 (Table 1). Compounds 5b and 2a gave typical fragmentation pattern at *m/z* 269 [M−H−146−H_2_O]^−^ (loss of moiety of O-linked rhamnose) and *m/z* 287 [M−H−146]^−^ (loss of a deoxyhexose moiety), characteristic of eriodictyol deoxyhexose derivatives [40]. Compounds 4d generated MS^2^ fragments at *m/z* 271 [M−H−162]^−^ indicating a loss of caffeoyl moiety, and according to the literature [17,21,33], this compound was assigned to naringenin-7-O glucoside. Compound 1c gave a [M−H]^−^ at *m/z* 271.1. This compound was tentatively characterized as naringenin based on its MS^2^ fragmentation at *m/z* 151 [M−H−120]^−^ indicating the presence of a C-hexosyl unit [33].

Three flavones, compound 10c (t_R_ = 15.30 min), compound 10a (t_R_ = 18.94 min), and compound 3d (t_R_ = 9.57 min) were respectively identified in *T. tetraptera*, *A. citratum,* and *X. parviflora* extracts (Figure S1A,C,D). Compounds 10c showed a [M−H]^−^ at *m/z* 359.2 with a loss of ethoxy group (245 [M−H−C_2_H_2_O]^−^) (Table 1), corresponding to the fragmentation of a trihydroxyflavone isomer. Compound 10a was tentatively assigned as a 5,7 dimethoxyflavone isomer with a deprotonated molecule at *m/z* 281.3 (Table 1) [41], while compound 3d gave a [M−H]^−^ at *m/z* 563.3 corresponding to apigenin 7-O-apiosyl-glucoside [42]. This was confirmed by the presence of fractions at *m/z* 383 [M−H−162−18]^−^ with the loss of an O-hexosyl moiety and a molecule of water, and at *m/z* 329 [M−H−269−60]^−^, corresponding to the deprotonation of an apigenin residue (Table 1).

Compound 9c (t_R_ = 14.18 min), compound 9a (t_R_ = 18.75 min), compound 7d (t_R_ = 14.05 min), with [M−H]^−^ at *m/z* 726.4, *m/z* 255.5, *m/z* 465.2 respectively (Figure S1A,C,D), were identified as anthocyanin, dihydroflavonol, and flavanonols compounds by comparison of their chromatographic retention times and MS^2^ fragmentation spectra. Compound 9c (Table 1) exhibited a fragment ion at *m/z* 656 [M−H−CO−C_2_H_2_O]^−^ and suggests the characteristic fragmentation of cyanidin 3-xylosylrutinoside isomer [43]. Compound 7d (Table 1) produced MS^2^ ions at *m/z* 319 [M−H−146]^−^ and 377 [M−H−2CO_2_]^−^ corresponding to the loss of moiety of O-linked rhamnose and a carboxyl group, was tentatively assigned taxifolin hexoside isomer [33].

#### 3.1.3. Other Compounds

In addition to the phenolic compounds described above, an alkylmethoxyphenol, compound 6b ([M−H]^−^ at *m/z* 221.1, t_R_ = 12.63 min), and a phytolaccagenic acid isomer, compound 3a ([M−H]^−^ at *m/z* 809.3, t_R_ = 11.94 min) were also detected in *D. glomerata* and *A. citratum* extracts, respectively (Figure S1A,B). The above compounds 6b and 3a respectively produced MS^2^ spectra with losses of water molecules and aliphatic residues (Table 1), which complied with the published literature [21,44]. No tentative identification could be proposed for compounds 6a ([M−H]^−^ at *m/z* 566.4, t_R_ = 15.91 min), 8a ([M−H]^−^ at *m/z* 279.1, t_R_ = 18.18 min), 7b ([M−H]^−^ at *m/z* 704.5, t_R_ = 14.25 min), 2c ([M−H]^−^ at *m/z* 1107.6, t_R_ = 11.86 min), 3c ([M−H]^−^ at *m/z* 1191.7, t_R_ = 12.03 min), 4c ([M−H]^−^ at *m/z* 1189.7, t_R_ = 12.22 min), 6c ([M−H]^−^ at *m/z* 820.4, t_R_ = 13.14 min), 12c ([M−H]^−^ at *m/z* 758.6, t_R_ = 20.02 min), 1d ([M−H]^−^ at *m/z* 374.1, t_R_ = 7.39 min), and 5d ([M−H]^−^ at *m/z* 333.2, t_R_ = 12.46 min), since it was not possible to assign the obtained ion fragments.

**Table 1:** Identification of polyphenol compounds in hydro-ethanolic extracts through UPLC-ESI-HRMS/MS analysis.

Peak No	(RT) (min)	[M–H]^-^ (*m/z*)	Fragment Ions	Tentative Identification (References)
Compound fractions from *Aframomum citratum* (C.Pereira) K.Schum				
1a	11.594	187.1	125.1045, 169.0948, 186.5114,187.1070.	Gallic acid monohydrate [33]
2a	11.722	433.1	433.1337, 269.0577, 152.0185, 259.0729, 287.0682.	Eriodictyol rhamnoside [40]
3a	11.941	809.3	165.0630, 175.0473, 176.0558, 191.0805, 405.1739.	Isomer of phytolaccagenic acid [21,44]
4a	15.255	315.3	141.1357, 279.2450, 297.2568, 313.2524, 315.2688.	Protocatechuic acid 4-O-glucoside [21,34]
5a	15.755	295.2	171.1097, 195.1476, 277.2295, 294.2950, 295.2412.	p-Coumaroyl tartaric acid derivative [17,21]
6a	15.910	566.4	224.0794, 242.0891, 281.2615, 506.3491, 566.3742.	Unknown
7a	17.553	473.3	109.0714, 311.0407, 357.3319, 385.3647, 429.3557, 473.3481.	Gallic acid derivative [34]
8a	18.181	279.1	223.1274, 278.3513, 279.458.	Unknown
9a	18.755	255.2	254.4282, 255.2440.	Dihydroxyflavanone derivative [36]
10a	18.949	281.3	280.3598, 281.2613.	Isomer of 5,7-dimethoxyflavone [41]
Compound fractions from *Dichrostachys glomerata* (Forssk.) Chiov				
1b	8.365	289.1	109.0356, 203.0802, 245.0925, 271.0730, 289.0849.	(+)-Catechin [33,38]
2b	8.943	561.2	245.0922, 289.0844, 271.0722, 407.0940, 561.1649.	Gallocatechin derivative [38]
3b	9.143	729.2	125.0309, 289.0844, 407.0948, 451.1226, 729.1808.	Procyanidin dimer monogallate [38,39]
4b	9.621	441.2	169.0216, 245.0924, 271.0730, 289.0846, 441.1005.	(Epi)catechin gallate [19]
5b	10.328	433.1	180.0144, 259.0720, 269.0570, 287.0682, 433.1322.	Eriodictyol rhamnoside [40]
6b	12.633	221.1	149.1036, 221.0931, 221.1284.	Alkylmethoxyphenol derivative [21,44]
7b	14.255	704.5	100.0458, 202.0810, 455.3721, 656.3367, 658.4625.	Unknown
8b	15.107	325.2	170.0116, 183.0205, 197.0362, 324,1567, 325.1992.	p-Coumaric acid 4-O-glucoside [17,21]
9b	16.058	311.2	148.6320, 149.1049, 310.2184, 311.2156.	Caftaric acid derivative [32]
Compound fractions from *Tetrapleura tetraptera* (Schum. and Thonn.) Taub				
1c	11.565	271.1	151.0110, 270.2059, 271.0739.	Naringenin [33]
2c	11.861	1107.6	657.4731, 817.5348, 961.5842, 979.5956, 1061.6415.	Unknown
3c	12.037	1191.7	655.4725, 817.5347, 961.5837, 979.5950, 1145.7018.	Unknown
4c	12.225	1189.7	491.3951, 563.4203, 815.5194, 959.5690, 977.5805.	Unknown
5c	12.545	841.5	441.3565, 603.4182, 751.500, 765.4799, 795.4926.	Proanthocyanidin trimer [37,38]
6c	13.142	820.4	628.4523, 684.3904, 686.4044, 730.3969, 774.3895.	Unknown
7c	13.492	865.5	455.3725, 640.4509, 658.4626, 776.5315, 820.5249.	Proanthocyanidin trimer [37,38]
8c	13.607	825.5	161.0527, 455.3738, 617.4362, 735.5049,779.4983.	Proanthocyanidin trimer [37,38]
9c	14.1867	726.4	100.0453, 656.3380, 658.4661, 726.4573.	Isomer of cyanidin 3-xylosylrutinoside [43]
10c	15.301	359.2	178.9161, 317.2262, 358, 0867, 359.2388.	Isomer of trihydroxyflavone [41]
11c	16.031	311.2	133.0731, 149.1050, 310.2188, 311.2661.	Caftaric acid derivative [32]
12c	20.029	758.6	89.0280, 119.0410, 532.4972, 550.5090, 712.5726.	Unknown
Compound fractions *from Xylopia parviflora* Spruce				
1d	7.395	374.1	166.0585, 207.0604, 328.1169, 374.1243, 374.2026.	Unknown
2d	8.413	289.1	203.0798, 205.0592, 245.0918, 271.0716, 289.0837.	(+)-Catechin [33,38]
3d	9.571	563.3	329.1557, 383.2043, 561.4585, 563.276.	Apigenin 7-O-apiosyl-glucoside [42]
4d	10.148	433.1	255.0398, 271.0352, 300.0391, 301.0474, 433.0944.	Naringenin hexoside [21,33]
5d	12.463	333.2	332.1515, 333.2203.	Unknown
6d	12.583	335.2	334.1605, 335.2356.	Caffeoylshikimic acid [35]
7d	14.052	465.2	318.2117, 319.2397,377.1922, 463.6790, 465.1749.	Isomer of taxifolin hexoside [33]
8d	14.376	315.2	314.1987, 315.2095, 315.2620.	Isomer of isorhamnetin [33]
9d	15.332	505.2	300.5674, 359.2367, 373.1619, 417.1890, 461.1799, 505.1717.	Quercetin 7-O-pentosyl-glucoside [45]
10d	15.652	339.2	170.0111, 183.0200, 197.0361, 338.1263, 339.2138.	Caffeoyl tricarboxylic acid isomer [33]
11d	16.964	301.2	300.2623, 301.0084, 301.2291.	Isomer of ellagic acid [36]

### 3.2. Quantitative Determination of Some Phenolic Compounds in Extracts

Phenolic compounds have always been an integral part of the secondary metabolites found in most plant extracts. During phenolic profiling by RP-HPLC, the presence of seven phenolic compounds was observed in the extracts: the most represented in each plant were underlined by statistical analysis (Table 2). The qualitative composition, with regards to the selected phenols, was only partially similar among plants. One phenolic acid, i.e., caffeic acid (7.07 ± 1.45 µg/100 mg of extract) was determined in *X. parviflora* extract. In addition, two flavanols, i.e., catechin (2.95 ± 0.70 µg/100 mg of extract) and epicatechin (106.91 ± 0.67 µg/100 mg of extract) were identified, with epicatechin significantly (*p* ≤ 0.05) higher than the two other compounds. According to the present study, it was reported in other recent papers [14] the presence of catechin compounds (amount of 3.31%) in the *X. parviflora* extract using GC-MS analysis. In *A. citratum*, three phenolic acids, i.e., protocatechuic acid (31.12 ± 0.96 µg/100 mg of extract), p-coumaric acid (8.49 ± 1.66 µg/100 mg of extract), rosmarinic acid (4.62 ± 0.15 µg/100 mg of extract) and kaempferol (111.79 ± 0.22 µg/100 mg of extract), were significantly (*p* ≤ 0.05) higher than the other compounds. [17] reported that the p-coumaric acid identified in this study was exactly 4-O glucoside p-coumaric acid. As well as in *A. citratum*, *T. tetraptera* contained kaempferol (13.96 ± 0.90 µg/100 mg of extract). Only two phenolic acids i.e., p-coumaric acid (22.39 ± 1.23 µg/100 mg of extract) and rosmarinic acid (22.03 ± 0.13 µg/100 mg of extract) were identified in *D. glomerata*. In comparison with the UPLC-ESI-HRMS/MS analysis of *D. glomerata* extract, no catechin molecules were found. It was therefore interesting to note that even other bioactive compounds were detectable and quantified.

### 3.3. FTIR Analysis of Extracts

The wavenumber ranges of the FTIR peaks and functional groups of each extract were determined by comparing them to the previous reports [46,47,48] (Table 3). The FTIR spectrum of *A. citratum, D. glomerata, T. tetraptera* and *X. parviflora* extracts (Figure S2A–D) showed absorption signals for 12 wavenumber or wavenumber ranges, which were identified as components in the samples namely phenols at 3276 cm^−1^, 3233 cm^−1^, 3283 cm^−1^, 3232 cm^−1^ (O–H), lipids at 1710–1733 cm^−1^, 1724 cm^−1^ (C=O), proteins at 1693 cm^−1^ (C=O, C–N), polysaccharides and carbohydrates at 2853–2924 cm^−1^, 2927 cm^−1^, 2928 cm^−1^, 2928 cm^−1^ (Csp^3^–H (CH_2_–H)), phenyl groups at 1443–1601 cm^−1^, 1604 cm^−1^, 1602 cm^−1^, 1603 cm^−1^ (C=C), amino acids at 1377 cm^−1^, 1372–1518 cm^−1^, 1372–1518 cm^−1^ (N–H, C–N), acids or esters at 1153–1238 cm^−1^, 1143–1284 cm^−1^, 1157–1236 cm^−1^ (Csp^2^–O (O–C=O or O–C–O)), alcohols at 1036 cm^−1^, 1040 cm^−1^, 1030 cm^−1^, 1065 cm^−1^ (Csp^3^–O (C–OH)), aromatic compounds at 674–867 cm^−1^, 767–867 cm^−1^, 776–886 cm^−1^, 663–886 cm^−1^ (cis C–H), isoprenoids at 420–617 cm^−1^, 423–636 cm^−1^, 419–630 cm^−1^, 427–626 cm^−1^ (cis C–H). Among these absorption signals, the FTIR spectra of *T. tetraptera* and *X. parviflora* extracts (Figure S2A,D) showed specific wavenumber ranges, namely aromatic secondary amines at 1259–1371 cm^−1^ (C–N) and mono–, oligo– carbohydrates, oligosaccharides, and glycoproteins at 926 cm^−1^, 931–979 cm^−1^ (*trans* C–H, Csp^3^–O (C–OH)). However, there were four signals, in which their function groups were still unknown, i.e., 1918–2350 cm^−1^, 1917–2350 cm^−1^, 1918–2350 cm^−1,^ and 1768–2326 cm^−1^. In corresponding previous reports, it has been reported that hydro-ethanolic extracts from the plants used in this study contained phenols, lipids, polysaccharides (generally in the form of glycosides), and aromatic compounds which are also consistent with the results obtained by the UPLC-ESI-HRMS/MS analysis [14,17,18]. Furthermore, these results may be due to the different solvation properties of ethanol and water.

### 3.4. Polyphenol Estimation (TPC, TFC, and FC Content) and Antioxidant Activities (DPPH and ABTS) of Extracts

The polyphenol content in each extract was measured as TPC, TFC, and FC (Table 4) and the results are expressed as gallic acid, catechin, and quercetin equivalents, respectively. Among the extracts, *D. glomerata* and *X. parviflora* possessed the highest TPC, with 282.62 ± 3.88 and 271.18 ± 7.10 mg gallic acid equivalents (GAE)/g respectively, followed by *T. tetraptera* (150.33 ± 0.036 mg gallic acid equivalent (GAE)/g) and *A. citratum* (129.36 ± 2.13 mg gallic acid equivalent (GAE)/g) [18,49] reported similarities and discrepancies between their findings on the same plants compared to this study. This may arise from different experimental protocols (i.e., organic solvent extraction vs. aqueous extraction) or the harvest period of plant material [16]. A similar trend, but with lower values in TFC was observed in each extract with 35.43 ± 1.33, 114 ± 1.32, 48.73 ± 4.38, 96.61 ± 0.86 mg catechin equivalents (CE)/g for *A. citratum*, *D. glomerata*, *T. tetraptera*, and *X. parviflora* respectively, indicating that the majority of phenolics present in extracts are flavonoids. Regarding the FC estimation, extracts showed values with a range of 0.58 to 0.21 mg quercetin equivalent (QE)/g, *D. glomerata* values (0.58 ± 0.14 mg quercetin equivalent (QE)/g) significantly (*p* ≤ 0.05) higher than the others. This study was similar and comparable with several studies on cultivars, including mango, blueberry, strawberry, raspberry, grapes, garlic, and ginger previously reported by [19,21,50].

The antioxidant capacities of each extract were evaluated through DPPH and ABTS methods. As shown in Table 4, *D. glomerata* showed the highest ABTS free radical scavenging activity (IC_50_ = 5.28 µg/mL) with 51.57 ± 0.74 mg Trolox equivalent (TE)/g of extract. *D. glomerata* was respectively followed by *X. parviflora* (IC_50_ = 14.01 µg/mL, 47.06 ± 1.05 mg TE/g of extract), *T. tetraptera* (IC_50_ = 28.15 µg/mL, 60.47 ± 1.05 mg TE/g of extract) and *A. citratum* (IC_50_ = 29.21 µg/mL, 52.66 ± 2.66 mg TE/g of extract). As previously noted, *D. glomerata* showed the highest DPPH antioxidant potential (IC_50_ = 15.06 µg/mL) with 254.30 ± 0.15 mg ascorbic acid equivalent (AAE)/g of extract, followed by *X. parviflora* (IC_50_ = 20.38 µg/mL, 253.54 ± 1.88 mg AAE/g of extract), *A. citratum* (IC_50_ = 41.04 µg/mL, 184.88 ± 0.14 mg AAE/g of extract) and *T. tetraptera* (IC_50_ = 45.67 µg/mL, 218.08 ± 1.20 mg AAE/g of extract). In both ABTS and DPPH assays, *D. glomerata* and *X. parviflora* extracts showed higher antioxidant potential when compared to the others. This could be due to a greater amount of antioxidant compounds (i.e., phenolic and flavonoid) contained in those extracts that likely could contribute to a strong antioxidant potential [51]. In addition, it is known that the level of antioxidant activity depends on the type and concentration of the phenolic compounds present because their structures greatly affect their bioactivity. Similar experimental procedures and findings were also reported by [49,52,53,54] when investigating the antioxidant activities of some dietary plants.

### 3.5. Correlations of Antioxidant Assays with Phenolic Compounds

The correlations between the phenolic estimation and antioxidant assays were carried out using the principal component analysis (PCA) and Pearson’s correlation test as shown in the supplementary data (Figure S3 and Table S1). The total phenolic acids and flavonoids were calculated by summing the proposed compounds from the HPLC-PDA quantitation to provide an idea of the overall correlations between all phenolic compounds and antioxidant tests. Polyphenols not found in the HPLC-PDA were not considered. Here, 90.16% variability of the initial data was kept by the first two main components (PC1 = 51.61% and PC2 = 38.52%, respectively) (Figure S3). DPPH and ABTS were highly correlated (*p* ≤ 0.05) to both TPC and TFC with positive correlation coefficients of R ≥ 0.96 (Table S1). These strong correlations suggested that polyphenols were the major contributors to the scavenging activity of the plant extracts. Moreover, the correlation between TPC and TFC parameters (R > 0.9, *p* ≤ 0.05) indicated that flavonoids were the main antioxidant polyphenols. Similar findings were also reported by [18,19,21,55] who studied the polyphenolic composition of some spice extracts. Furthermore, it is observed that DPPH and ABTS tests were positively correlated since Pearson’s correlation coefficient was R = 0.97 (*p* ≤ 0.05) between the assays (Table S1), in line with previous studies [56,57,58]. Since DPPH is only applicable to hydrophobic systems due to the use of a radical dissolved in organic media, the strong correlation with ABTS indicated that additional and less hydrophilic compounds may also contribute to the scavenging effect [59]. On the contrary, the HPLC-detected phenolic acids were negatively correlated (*p* ≤ 0.05) with the antioxidant assays (Table S1), suggesting that within the selected samples in this study, the phenolic acids do not significantly contribute to the antioxidant activities, maybe because of vitamins (carotenoids, vitamin C, vitamin E) and antioxidant heteropolysaccharides and polypeptides.

### 3.6. Cytotoxicity and Effect of the MIX on the TNFα-Induced NF-κB Driven Transcription in AGS and GES-1 Cells

**Figure 1:**
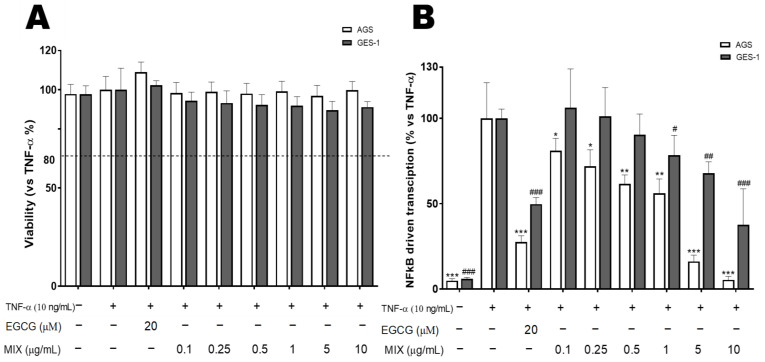
Cytotoxicity of the extracts in combination (MIX) (**A**) and their effect on the NF-κB-driven transcription in human gastric adenocarcinoma (AGS) and gastric epithelial (GES-1) cells (**B**). Data are expressed as percentages versus the stimulated control, which is arbitrarily set to 100%. * *p* < 0.05; ** *p* < 0.01; *** *p* < 0.001 in AGS cells and ^#^ *p* < 0.05, ^##^ *p* < 0.01 and ^###^ *p* < 0.001 in GES−1 cells. EGCG: Epigallocatechin-3-O-gallate, +: Present, −: Absent.

These studies were performed using two models of gastric epithelial cells (AGS and GES-1 cells) (Figure S4) stimulated with TNFα (10 ng/mL), a cytokine that contributes to the inflammatory process during gastric epithelium infection. The cytotoxicity of extracts in combination (namely MIX) was assessed in the concentration range of 0.1–10 µg/mL through the MTT assay (Figure 1A). Similar to our earlier study [5], in which extracts were used as a single treatment, no cytotoxic effects of extracts in combination were observed in the cell cultures after 6 h treatment (Figure 1A). Extracts in combination (MIX) were investigated for their ability to inhibit the TNFα-induced NF-κB driven transcription in a concentration-dependent manner (Figure 1B). The MIX was tested in the range of 0.1–10 µg/mL and the corresponding IC_50_ values were calculated (Table 5). In AGS cells, the MIX significantly impaired the activation of NF-κB driven transcription with more than 95% reduction (*p* ≤ 0.001) observed at 10 µg/mL and IC_50_ (0.7 µg/mL) below the previously reported IC_50_s (2.14–9.67 µg/mL) for each plant [5]. In GES-1 cells, the MIX at concentrations ranging between 5 and 10 µg/mL, significantly (*p* ≤ 0.01) impaired the activation of NF-κB driven transcription (32.14–62.39% reduction). The IC_50_ (4.9 µg/mL) value was higher compared to the one obtained in AGS cells, but below IC_50_s (5.22–12.17 µg/mL) for each plant summarized in Table 5. The treatment with the reference compound (20 μM EGCG) yielded a significant (*p* ≤ 0.001) inhibition of NF-κB driven transcription in AGS (>70% reduction) and GES-1 (>50% reduction) cells, which was higher than the MIX at concentrations below 1 µg/mL). Moreover, the basal concentration of the NF-κB driven transcription was not affected by the extracts used as single or in combination (all tested at 10 µg/mL) (Figure S5). The chemical analysis of each extract reported the presence of many groups of compounds (polyphenols, carbohydrates, lipids, and proteins). Based on this characterization, these metabolites can act as NF-κB inhibitors, such as epigallocatechin, quercetin, cyanidin, chlorogenic acid, and catechins, identified in some extracts. Several studies reported that phenolic acids stimulate the inhibition of NF-κB activation and macrophage infiltration, resulting in the reduction of inflammation in vitro and in animal models [60,61,62,63]. In addition to their role in food intake regulation and nutrition absorption, a growing body of evidence supports that flavonoids counteract NF-κB and inducible nitric oxide synthase (iNOS) signaling pathways, resulting in reduced oxidative damage and inflammation [64,65].

### 3.7. The Combination of Extracts (MIX) Inhibits TNFα-Induced IL-8 and IL-6 Release in AGS and GES-1 Cells

**Figure 2:**
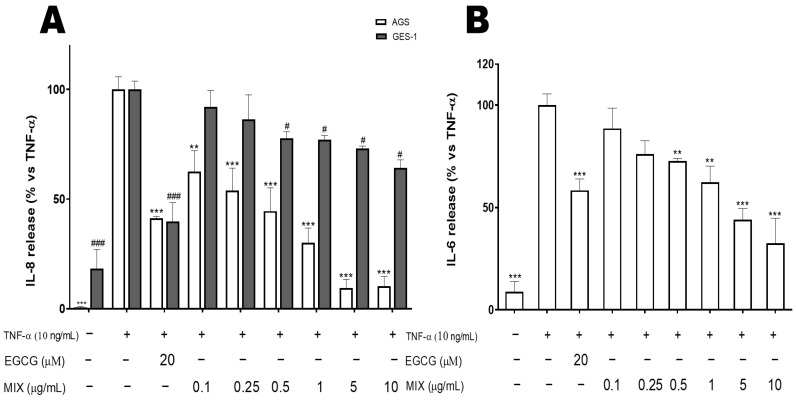
Effect of the MIX on the TNFα-Induced IL-8 (**A**) and IL-6 (**B**) release in human gastric adenocarcinoma (AGS) and gastric epithelial (GES-1) cells. Data are expressed as percentages versus the stimulated control, which is arbitrarily set to 100%. ** *p* < 0.01; *** *p* < 0.001 in AGS cells and ^#^ *p* < 0.05, and ^###^*p* < 0.001 in GES-1 cells. EGCG: Epigallocatechin-3-O-gallate, +: Present, −: Absent.

IL-8 and IL-6 are well-known NF-κB-dependent cytokines that are implicated in the gastric inflammatory process [66,67]. The MIX at concentrations ranging between 0.1 and 10 µg/mL, inhibited IL-8 release in AGS and GES-1 cells with different IC_50_s (Figure 2A, Table 5). In AGS cells, the MIX was more active at 5 and 10 µg/mL (the highest concentrations tested) compared to the reference compound (20 μM EGCG, >58% reduction) and significantly (*p* ≤ 0.001) inhibited the release of IL-8 (90% reduction) with an IC_50_ (0.27 µg/mL) below the one of *A. citratum* (0.35 µg/mL), *T. tetraptera* (1.37 µg/mL), *X. parviflora* (0.30 µg/mL), but over the IC_50_ of *D. glomerata* (0.29 µg/mL) used as single. This value was lower in AGS cells compared to GES-1 cells (IC_50_ > 10 µg/mL), as noted earlier in the NF-κB driven transcription results. However, the IC_50_s of the extracts used as single were lower in GES-1 cells (2.30–8.37 µg/mL) in comparison to the IC_50_ of the MIX. In this study, no detectable IL-6 secretion was induced in the AGS cells (data not shown); thus, only GES-1 cells were considered. The MIX also inhibited the IL-6 release in GES-1 cells with an IC_50_ of 1.8 µg/mL (Figure 2B, Table 5), a value which was below the IC_50_s (3.47–5.11 µg/mL) of the extracts used as single. In addition, as noted in AGS cells, the MIX was more active at 5 and 10 µg/mL (the highest concentrations tested) compared to the reference compound (20 μM EGCG, around 40% inhibition). This suggests that it can reduce the release and gene expression of NF-κB-dependent pro-inflammatory mediators, which contribute to the amplification of the gastric inflammatory process.

### 3.8. Synergistic Effect of Extracts in Combination on AGS and GES-1 Cells

Combined therapy has shown a variety of advantages over monotherapy, including decreasing the concentration and toxicity of drugs, improving efficiency, targeting multiple molecular pathways, and sensitizing cells to treatment [68]. As the above statements make clear, the definition of synergy can overlap with potentiation. However, a concrete definition derives only from a mathematical approach, shown and proven by different methodologies, such as Berenbaum’s pioneering work, the isobologram method of Loewe, the fractional product method of Webb, the combination index method of Chou and Talalay, which provided the foundation for its use in pharmacology and phytopharmacology [69]. Therefore, in the current study, we tested the synergistic effects of the extracts on inflammatory markers involved in gastric inflammation. The effects of each extract (used as a single) on the TNFα-induced cytokines (IL-8, IL-6) release and NF-κB driven transcription of AGS and GES-1 cells, previously reported by Nwakiban et al. [5] and their combination are shown in Table 5. The CI values of IL-8, IL-6 release, and NFκB-driven transcription were more than one (6.18) in GES-1 cells and 0.82 (slight synergism) in AGS cells; 0.46 (synergism) in GES-1 cells; 0.61 (synergism) in GES-1 cells and 0.18 (strong synergism) in AGS cells respectively, which in overall indicates a synergistic effect of the MIX on cells against the gastric inflammatory process. Moreover, the means DRI of extracts in combination were 0.80 ± 0.40 in GES-1 and 8.18 ± 8.13 in AGS cells for IL-8 release; 9.22 ± 1.91 in GES-1 cells for IL-6 release; 14.28 ± 4.6 in GES-1 and 28.74 ± 11.58 in AGS cells for NFκB driven transcription, which suggests a very high-fold dosage reduction compared to each extract in monotherapy, i.e., eight-fold dosage reduction for IL-8 release in AGS cells and fourteen-fold dosage reduction for the NF-κB driven transcription in GES-1 cells. the MIX has been noted to act antagonistically in GES-1 cells on NF-κB transcription (CI > 1), however, it was found to exert an NF-κB inhibition activity in a concentration-dependent fashion (Figure 1B). Overall, the biological activities of the MIX were lower in AGS cells compared to GES-1 cells as was previously reported [5]. This could be attributed to the differences in mutation and differentiation of GES-1 and AGS cells, but also due to the differences in their genetic profiles [31]. Furthermore, the natural bioactive compounds found in each extract have a more structural diversity that could more effectively inhibit certain targets of gastric disorders (gene expression of proinflammatory cytokines). They also inherently target other biologically relevant NF-κB pathways, i.e., enzymes such as prostaglandin-endoperoxide synthase 2 (PTGS2) (COX-2), because many natural bioactive compounds are secondary metabolites or signaling molecules [5,70]. Based on the above findings, it is suggested that the MIX contains various types of bioactive compounds that act in synergy in human gastric epithelial cells by two or more mechanisms (synergistic multi-target effects and elimination or neutralization potential).

## 4. Conclusions

In this study, a comparative analysis of the chemical composition of each hydroalcoholic extract through RP-HPLC, UPLC-ESI-HRMS/MS, and FTIR indicates that they mostly contain a great number of phenolic compounds, but also proteins, lipids, polysaccharides (generally in the form of glycosides) and aromatic compounds. The extracts showed a high amount of total phenolic (TPC: 150–290 mg GAE/g of extract) and flavonoid content (TFC: 35–115 mg CE/g of extract) with antioxidant properties in a cell-free system (DPPH IC_50_s ≤ 45 µg/mL; ABTS IC_50_s ≤ 29 µg/mL). The extracts in combination (MIX) exert a synergistic beneficial effect (CIs < 1 and DRIs > 1) on inflammatory markers (IL-8, IL-6 release, and NF-κB driven transcription) in human gastric epithelial cells which may be due to the presence of phenolic compounds (mostly phenolic acids and flavonoids). Among the phenolic compounds, phenolic acids (hydroxybenzoic and hydroxycinnamic acids) and flavonoids (anthocyanins, flavanols, flavanonols, flavanones, flavones, flavonols, and isoflavonoids) which promoted an antioxidant potential, have been reported in the extracts. The MIX enhances the efficacy of extract used in monotherapy and reduces the potential adverse effects related to high concentrations, as indicated by high DRI. Beyond the plausible pharmacodynamic interaction among phenolic compounds, further studies are required to assess other possible mechanisms of synergistic interaction, and to in vivo confirm the synergistic effect herein reported. To the best of our knowledge, this study provides a scientific basis for the traditional practice of using a combination of extracts useful to alleviate gastric inflammation.

## Figures and Tables

**Table 2:** Phenolic (µg/100 mg of extract) content in each extract through HPLC-PDA analysis.

Phenolic Compounds	*Aframomum citratum*	*Dichrostachys glomerata*	*Tétrapleura tetraptera*	*Xylopia parviflora*	λmax (nm)
Protocatecheuic acid	31.12 ± 0.96 ^b^	-	-	-	280
Epicatechin	-	-	-	106.91 ± 0.67 ^b^	280
Catechin	-	-	-	2.95 ± 0.70 ^a^	320
Caffeic acid	-	-	-	7.07 ± 1.45 ^a^	320
p-Coumaric acid	8.49 ± 1.66 ^a^	22.39 ± 1.23 ^a^	-	-	320
Rosmarinic acid	4.62 ± 0.15 ^a^	22.03 ± 0.13 ^a^	-	-	320
Kaempferol	111.79 ± 0.22 ^c^	-	13.96 ± 0.9	-	320

-: not found.; Data are expressed as mean ± standard deviation (*n* = 3). The values with different lowercase letters are significantly different at *p* ≤ 0.05 within each column.

**Table 3:** Functional groups and mode of vibration from FTIR spectra of extracts.

The Wavelength Range Found in This Study, cm^−1^)	Assignment of Bonds	Mode of Vibration	Function Groups
Functional groups and mode of vibration from FTIR spectra of *Aframomum citratum* (C.Pereira) K.Schum			
3276	O–H	O–H stretching	Phenols
2853–2924	Csp^3^–H (CH_2_–H)	CH_2_–H stretching	Polysaccharides, lipids, and carbohydrates
1918–2350	–	–	Unknown
1710–1733	C=O	C=O stretching	Lipids
1443–1601	C=C	C=C aromatic ring stretching	Phenyl groups
1377	N–H, C–N	N–H, C–N stretching	Amino acids
1153–1238	Csp^2^–O (O–C=O or O–C–O)	C–O stretching	Acids or esters
1036	Csp^3^–O (C–O)	C–O stretching	Alcohols
674–867	cis C–H	Aromatic C–H bending	Aromatic compounds
420–617	cis C–H	C–H bending	Isoprenoids
Functional groups and mode of vibration from FTIR spectra of *Dichrostachys glomerata* (Forssk.) Chiov			
3233	O–H	O–H stretching	Phenols
2927	Csp^3^–H (CH_2_–H)	CH_2_–H stretching	Polysaccharides, lipids, and carbohydrates
1917–2350	–	–	Unknown
1604	C=C	C=C– Aromatic ring stretching	Phenyl groups
1372–1518	N–H, C–N	N–H, C–N stretching	Amino acids
1143–1284	Csp^2^–O (O–C=O or O–C–O)	–C–O stretching	Acids or esters
1040	Csp^3^–O (C–OH)	C–O stretching	Alcohols
767–867	*cis* C–H	Aromatic C–H bending	Aromatic compounds
423–636	*cis* C–H	C–H bending	Isoprenoids
Functional groups and mode of vibration from FTIR spectra of *Tetrapleura tetraptera* (Schum. and Thonn.)Taub			
3283	O–H	O–H stretching	Phenols
2928	Csp^3^–H (CH_2_–H)	CH_2_–H stretching	Polysaccharides, lipids, and carbohydrates
1918–2350	–	–	Unknown
1693	C=O, C–N	C=O, C–N stretching	Proteins
1602	C=C	C=C– Aromatic ring stretching	Phenyl groups
1259–1371	C–N	C–N stretching	Aromatic secondary amines
1030	Csp^3^–O (C–OH)	C–O stretching	Alcohols
926	*trans* C–H, Csp^3^–O (C–OH)	*trans* C–H and Csp^3^–O (C–OH) stretching	Alcohols, mono–, oligo–carbohydrates, oligosaccharides, glycoproteins
776–886	*cis* C–H	Aromatic C–H bending	Aromatic compounds
419–630	*cis* C–H	C–H bending	Isoprenoids
Functional groups and mode of vibration from FTIR spectra of *Xylopia parviflora* Spruce			
3232	–O–H	O–H stretching	Phenols
2928	Csp^3^–H (CH_2_–H)	CH_2_–H stretching	Polysaccharides, lipids, and carbohydrates
1768–2326	–	–	Unknown
1724	C=O	C=O stretching	Lipids
1693	C=O, C–N	C=O, C–N stretching	Proteins
1603	C=C	C=C– aromatic ring stretching	Phenyl groups
1372–1518	N–H, C–N	N–H, C–N stretching	Amino acids
1157–1236	Csp^2^–O (O–C=O or O–C–O)	C–O stretching	Acids or esters
1065	Csp^3^–O (C–OH)	C–O stretching	Alcohols
931–979	*trans* C–H, Csp^3^–O (C–OH)	*trans* C–H and Csp^3^–O (C–OH) stretching	Alcohols, mono–, oligo–carbohydrates, oligosaccharides, glycoproteins
663–886	*cis* C–H	Aromatic C–H bending	Aromatic compounds
427–626	*cis* C–H	C–H bending	Isoprenoids

–: not found.

**Table 4:** Polyphenol content estimation and antioxidant activities of spice extracts.

Names of the Extracts	Polyphenol Estimation			Antioxidant Activities			
	TPC (mg GAE/g of Extract)	TFC (mg CE/g of Extract)	FC (mg QE/g of Extract)	ABTS		DPPH	
				mg TE/g of Extract	IC_50_ (µg/mL)	mg AAE/g of Extract	IC_50_ (µg/mL)
*A. citratum*	129.36 ± 2.13 ^a^	35.43 ± 1.33 ^a^	0.37 ± 0.02 ^a^	52.66 ± 2.66 ^a^	29.21	184.88 ± 0.14 ^a^	41.04
*D. glomerata*	282.62 ± 3.88 ^b^	114 ± 1.32 ^c^	0.58 ± 0.14 ^b^	51.57 ± 0.74 ^a^	5.28	254.30 ± 0.15 ^c^	15.06
*T. tetraptera*	150.33 ± 0.036 ^a^	48.73 ± 4.38 ^b^	0.26 ± 0.07 ^a^	60.47 ± 1.05 ^b^	28.15	218.08 ± 1.20 ^b^	45.67
*X. parviflora*	271.18 ± 7.10 ^b^	96.61 ± 0.86 ^c^	0.21 ± 0.04 ^a^	47.06 ± 1.05 ^a^	14.01	253.54 ± 1.88 ^c^	20.38

Data are expressed as mean ± standard deviation (*n* = 3). The values with different lowercase letters are significantly different at *p* ≤ 0.05 within each column. AAE: Ascorbic acid equivalents; ABTS: 2,2′-azinobis-(3-ethylbenzothiazoline-6-sulfonic acid); DPPH: 1,1-Diphenyl-2-picryl-hydrazyl; CE: Catechin equivalent; FC: Flavonol content; GAE: Gallic acid equivalent; IC_50_: Half maximal inhibitory concentration; QE: Quercetin equivalent; TFC: Total flavonoid content; TPC: Total phenol content; TE: Trolox equivalent.

**Table 5:** Half-maximal inhibitory concentrations (IC_50_) (µg/mL) as individual extracts [5] or a combination of the TNFα-induced cytokines release and NF-κB driven transcription in human gastric adenocarcinoma (AGS) and gastric epithelial (GES−1) cells.

Names of Plants	IL-8 Release IC_50_ (µg/mL)		IL-6 Release IC_50_ (µg/mL)	NFkB Driven Transcription IC_50_ (µg/mL)	
Half-maximal inhibitory concentrations (IC_50_s) summary of extracts used as single and in combination					
	GES-1	AGS	GES-1	GES-1	AGS
*Aframomum citratum* (C.Pereira) K.Schum	2.30	0.35	5.11	9.08	6.80
*Dichrostachys glomerata* (Forssk.) Chiov.	4.20	0.19	3.50	8.79	2.14
*Tetrapleura tetraptera* (Schum. and Thonn.)Taub	5.71	1.37	4.90	5.22	9.67
*Xylopia parviflora* Spruce	8.37	0.30	3.47	12.17	4.10
Combination of extracts (MIX)	>10	0.27	1.8	4.9	0.7
Combination and dose reduction indices of interaction between extracts					
	GES-1	AGS	GES-1	GES-1	AGS
Combination Index (CI) of the MIX	6.18	0.82 ^++^	0.46 ^+++^	0.61 ^+++^	0.18 ^++++^
Means of dose reduction index (DRI) of extracts	0.80 ± 0.40	8.18 ± 8.13	9.22 ± 1.91	14.28 ± 4.6	28.74 ± 11.58

The means of the dose reduction index (DRI) of extracts are expressed as mean ± standard deviation (*n* = 4). The graded symbols indicate ^++^: Moderate synergism (0.7 < CI < 0.85), ^+++^: Synergism (0.3 < CI < 0.7), ^++++^: Strong synergism (0.1 < CI < 0.3). Experiments using individual or a combination of extracts were performed at the same time.

## Data Availability

The data presented in this study are available in the article and Supplementary Materials.

## Supplementary Materials

The following supporting information can be downloaded at: https://www.mdpi.com/article/10.3390/antiox12030591/s1, Figure S1: UPLC-ESI-MS/MS mass spectra of *Aframomum citratum* (C.Pereira) K.Schum (A), *Dichrostachys glomerata* (Forssk.) Chiov. (B), *Tetrapleura tetraptera* (Schum. and Thonn.)Taub (C) and *Xylopia parviflora* Spruce (D) extracts; Figure S2: FTIR spectra of *Aframomum citratum* (C.Pereira) K.Schum (A), *Dichrostachys glomerata* (Forssk.) Chiov. (B), *Tetrapleura tetraptera* (Schum. and Thonn.)Taub (C) and *Xylopia parviflora* Spruce (D) extracts; Figure S3: Principal component analysis (PCA) of the antioxidant assays, phenolic acids, and flavonoids determined by HPLC-PDA; Figure S4: Microscopy (inverted phase contrast microscopy; 10X magnification) representation of human normal gastric (GES-1) cells (A) gastric adenocarcinoma (AGS) (B) epithelial cells; Figure S5: Effect of extracts in monotherapy and combination on the basal level of NF-κB-driven transcription in non-stimulated human gastric adenocarcinoma (AGS) and gastric epithelial (GES-1) cells. Table S1: Correlation coefficient (R) among the assays (antioxidant assays with phenolic compounds).

## References

[1] Chow D.K.L., Sung J.J.Y. (2009). Non-NSAID Non-H. Pylori Ulcer Disease. Best Pract. Res. Clin. Gastroenterol..

[2] de Barros M., Mota da Silva L., Boeing T., Somensi L.B., Cury B.J., de Moura Burci L., Santin J.R., de Andrade S.F., Monache F.D., Cechinel-Filho V. (2016). Pharmacological Reports about Gastroprotective Effects of Methanolic Extract from Leaves of *Solidago chilensis* (Brazilian Arnica) and Its Components Quercitrin and Afzelin in Rodents. Naunyn-Schmiedeberg’s Arch. Pharm..

[3] Mota da Silva L., Boeing T., Somensi L.B., Cury B.J., Bispo Steimbach V.M., de Oliveira Silveria A.C., Niero R., Cechinel Filho V., Santin J.R., de Andrade S.F. (2015). Evidence of Gastric Ulcer Healing Activity of Maytenus Robusta Reissek: In Vitro and in Vivo Studies. J. Ethnopharmacol..

[4] Martinelli G., Angarano M., Piazza S., Fumagalli M., Magnavacca A., Pozzoli C., Khalilpour S., Dell’Agli M., Sangiovanni E. (2022). The Nutraceutical Properties of Sumac (*Rhus coriaria* L.) against Gastritis: Antibacterial and Anti-Inflammatory Activities in Gastric Epithelial Cells Infected with *H. pylori*. Nutrients.

[5] Nwakiban A.P.A., Fumagalli M., Piazza S., Magnavacca A., Martinelli G., Beretta G., Magni P., Tchamgoue A.D., Agbor G.A., Kuiaté J.-R. (2020). Dietary Cameroonian Plants Exhibit Anti-Inflammatory Activity in Human Gastric Epithelial Cells. Nutrients.

[6] AL-Wajeeh N.S., Hajerezaie M., Noor S.M., Halabi M.F., Al-Henhena N., Azizan A.H.S., Kamran S., Hassandarvish P., Shwter A.N., karimian H. (2017). The Gastro Protective Effects of Cibotium Barometz Hair on Ethanol-Induced Gastric Ulcer in Sprague-Dawley Rats. BMC Vet. Res..

[7] Adzu B., Balogun S.O., Pavan E., Ascêncio S.D., Soares I.M., Aguiar R.W.S., Ribeiro R.V., Beserra Â.M.S.E.S., de Oliveira R.G., da Silva L.I. (2015). Evaluation of the Safety, Gastroprotective Activity and Mechanism of Action of Standardised Leaves Infusion Extract of *Copaifera malmei* Harms. J. Ethnopharmacol..

[8] Awaad A.S., El-Meligy R.M., Soliman G.A. (2013). Natural Products in Treatment of Ulcerative Colitis and Peptic Ulcer. J. Saudi Chem. Soc..

[9] Fumagalli M., Sangiovanni E., Vrhovsek U., Piazza S., Colombo E., Gasperotti M., Mattivi F., De Fabiani E., Dell’Agli M. (2016). Strawberry Tannins Inhibit IL-8 Secretion in a Cell Model of Gastric Inflammation. Pharmacol. Res..

[10] Sangiovanni E., Piazza S., Vrhovsek U., Fumagalli M., Khalilpour S., Masuero D., Di Lorenzo C., Colombo L., Mattivi F., De Fabiani E. (2018). A Bio-Guided Approach for the Development of a Chestnut-Based Proanthocyanidin-Enriched Nutraceutical with Potential Anti-Gastritis Properties. Pharmacol. Res..

[11] Cross L.B., Justice L.N. (2002). Combination Drug Therapy for Gastroesophageal Reflux Disease. Ann. Pharmacother..

[12] Fisher A.A., Le Couteur D.G. (2001). Nephrotoxicity and Hepatotoxicity of Histamine H2 Receptor Antagonists. Drug Saf..

[13] Nwakiban A.P.A., Sangiovanni E., Piazza S., Fumagalli M., Beretta G., Agbor G.A., Kuiaté J.-R., Dell’Agli M. (2019). Nutritional spices from Cameroon inhibit inflammatory markers from human gastric epithelial cells. Planta Med..

[14] Atchan Nwakiban A.P., Sokeng A.J., Dell’Agli M., Bossi L., Beretta G., Gelmini F., Deutou Tchamgoue A., Agbor Agbor G., Kuiaté J.-R., Daglia M. (2019). Hydroethanolic Plant Extracts from Cameroon Positively Modulate Enzymes Relevant to Carbohydrate/Lipid Digestion and Cardio-Metabolic Diseases. Food Funct..

[15] Atchan Nwakiban A.P., Passarelli A., Da Dalt L., Olivieri C., Demirci T.N., Piazza S., Sangiovanni E., Carpentier-Maguire E., Martinelli G., Shivashankara S.T. (2021). Cameroonian Spice Extracts Modulate Molecular Mechanisms Relevant to Cardiometabolic Diseases in SW 872 Human Liposarcoma Cells. Nutrients.

[16] Cicolari S., Dacrema M., Tsetegho Sokeng A.J., Xiao J., Atchan Nwakiban A.P., Di Giovanni C., Santarcangelo C., Magni P., Daglia M. (2020). Hydromethanolic Extracts from *Adansonia digitata* L. Edible Parts Positively Modulate Pathophysiological Mechanisms Related to the Metabolic Syndrome. Molecules.

[17] Nwakiban Atchan A.P., Shivashankara S.T., Piazza S., Tchamgoue A.D., Beretta G., Dell’Agli M., Magni P., Agbor G.A., Kuiaté J.-R., Manjappara U.V. (2022). Polyphenol-Rich Extracts of Xylopia and Aframomum Species Show Metabolic Benefits by Lowering Hepatic Lipid Accumulation in Diet-Induced Obese Mice. ACS Omega.

[18] Atchan Nwakiban A.P., Cicolari S., Piazza S., Gelmini F., Sangiovanni E., Martinelli G., Bossi L., Carpentier-Maguire E., Deutou Tchamgoue A., Agbor G.A. (2020). Oxidative Stress Modulation by Cameroonian Spice Extracts in HepG2 Cells: Involvement of Nrf2 and Improvement of Glucose Uptake. Metabolites.

[19] Gu C., Howell K., Dunshea F.R., Suleria H.A.R. (2019). LC-ESI-QTOF/MS Characterisation of Phenolic Acids and Flavonoids in Polyphenol-Rich Fruits and Vegetables and Their Potential Antioxidant Activities. Antioxidants.

[20] Peng D., Zahid H.F., Ajlouni S., Dunshea F.R., Suleria H.A.R. (2019). LC-ESI-QTOF/MS Profiling of Australian Mango Peel By-Product Polyphenols and Their Potential Antioxidant Activities. Processes.

[21] Tang J., Dunshea F.R., Suleria H.A.R. (2020). LC-ESI-QTOF/MS Characterization of Phenolic Compounds from Medicinal Plants (Hops and Juniper Berries) and Their Antioxidant Activity. Foods.

[22] Singleton V.L., Rossi J.A. (1965). Colorimetry of Total Phenolics with Phosphomolybdic-Phosphotungstic Acid Reagents. Am. J. Enol. Vitic..

[23] Zhishen J., Mengcheng T., Jianming W. (1999). The Determination of Flavonoid Contents in Mulberry and Their Scavenging Effects on Superoxide Radicals. Food Chem..

[24] Moukette B.M., Pieme C.A., Njimou J.R., Biapa C.P.N., Marco B., Ngogang J.Y. (2015). In Vitro Antioxidant Properties, Free Radicals Scavenging Activities of Extracts and Polyphenol Composition of a Non-Timber Forest Product Used as Spice: Monodora Myristica. Biol. Res..

[25] Horszwald A., Julien H., Andlauer W. (2013). Characterisation of Aronia Powders Obtained by Different Drying Processes. Food Chem..

[26] Kumaran A., Joel Karunakaran R. (2007). In Vitro Antioxidant Activities of Methanol Extracts of Five Phyllanthus Species from India. LWT-Food Sci. Technol..

[27] Sogi D.S., Siddiq M., Greiby I., Dolan K.D. (2013). Total Phenolics, Antioxidant Activity, and Functional Properties of ‘Tommy Atkins’ Mango Peel and Kernel as Affected by Drying Methods. Food Chem..

[28] Re R., Pellegrini N., Proteggente A., Pannala A., Yang M., Rice-Evans C. (1999). Antioxidant Activity Applying an Improved ABTS Radical Cation Decolorization Assay. Free Radic. Biol. Med..

[29] Sangiovanni E., Fumagalli M., Pacchetti B., Piazza S., Magnavacca A., Khalilpour S., Melzi G., Martinelli G., Dell’Agli M. (2019). *Cannabis sativa* L. Extract and Cannabidiol Inhibit in Vitro Mediators of Skin Inflammation and Wound Injury. Phytother. Res..

[30] Chou T.-C., Talalay P. (1984). Quantitative Analysis of Dose-Effect Relationships: The Combined Effects of Multiple Drugs or Enzyme Inhibitors. Adv. Enzym. Regul..

[31] Kamran S., Sinniah A., Chik Z., Alshawsh M.A. (2022). Diosmetin Exerts Synergistic Effects in Combination with 5-Fluorouracil in Colorectal Cancer Cells. Biomedicines.

[32] Carazzone C., Mascherpa D., Gazzani G., Papetti A. (2013). Identification of Phenolic Constituents in Red Chicory Salads (*Cichorium intybus*) by High-Performance Liquid Chromatography with Diode Array Detection and Electrospray Ionisation Tandem Mass Spectrometry. Food Chem..

[33] Kang J., Price W.E., Ashton J., Tapsell L.C., Johnson S. (2016). Identification and Characterization of Phenolic Compounds in Hydromethanolic Extracts of Sorghum Wholegrains by LC-ESI-MS(n). Food Chem..

[34] Álvarez-Fernández M.A., Cerezo A.B., Cañete-Rodríguez A.M., Troncoso A.M., García-Parrilla M.C. (2015). Composition of Nonanthocyanin Polyphenols in Alcoholic-Fermented Strawberry Products Using LC–MS (QTRAP), High-Resolution MS (UHPLC-Orbitrap-MS), LC-DAD, and Antioxidant Activity. J. Agric. Food Chem..

[35] Ben Said R., Hamed A.I., Mahalel U.A., Al-Ayed A.S., Kowalczyk M., Moldoch J., Oleszek W., Stochmal A. (2017). Tentative Characterization of Polyphenolic Compounds in the Male Flowers of Phoenix Dactylifera by Liquid Chromatography Coupled with Mass Spectrometry and DFT. Int. J. Mol. Sci..

[36] Fischer U.A., Carle R., Kammerer D.R. (2011). Identification and Quantification of Phenolic Compounds from Pomegranate (*Punica granatum* L.) Peel, Mesocarp, Aril and Differently Produced Juices by HPLC-DAD–ESI/MSn. Food Chem..

[37] Kajdžanoska M., Gjamovski V., Stefova M. (2010). HPLC-DAD-ESI-MSn Identification of Phenolic Compounds in Cultivated Strawberries from Macedonia. Maced. J. Chem. Chem. Eng..

[38] Rockenbach I.I., Jungfer E., Ritter C., Santiago-Schübel B., Thiele B., Fett R., Galensa R. (2012). Characterization of Flavan-3-Ols in Seeds of Grape Pomace by CE, HPLC-DAD-MSn and LC-ESI-FTICR-MS. Food Res. Int..

[39] Mata A., Ferreira J.P., Semedo C., Serra T., Duarte C.M.M., Bronze M.R. (2016). Contribution to the Characterization of Opuntia Spp. Juices by LC–DAD–ESI-MS/MS. Food Chem..

[40] Wu G., Bennett S.J., Bornman J.F., Clarke M.W., Fang Z., Johnson S.K. (2017). Phenolic Profile and Content of Sorghum Grains under Different Irrigation Managements. Food Res. Int..

[41] Pei K., Gui T., Kan D., Feng H., Jin Y., Yang Y., Zhang Q., Du Z., Gai Z., Wu J. (2020). An Overview of Lipid Metabolism and Nonalcoholic Fatty Liver Disease. BioMed Res. Int..

[42] Ibrahim R.M., El-Halawany A.M., Saleh D.O., Naggar E.M.B.E., El-Shabrawy A.E.-R.O., El-Hawary S.S. (2015). HPLC-DAD-MS/MS Profiling of Phenolics from *Securigera securidaca* Flowers and Its Anti-Hyperglycemic and Anti-Hyperlipidemic Activities. Rev. Bras. Farmacogn..

[43] Brito A., Ramirez J.E., Areche C., Sepúlveda B., Simirgiotis M.J. (2014). HPLC-UV-MS Profiles of Phenolic Compounds and Antioxidant Activity of Fruits from Three Citrus Species Consumed in Northern Chile. Molecules.

[44] Saleri F.D., Chen G., Li X., Guo M. (2017). Comparative Analysis of Saponins from Different Phytolaccaceae Species and Their Antiproliferative Activities. Molecules.

[45] Tsetegho Sokeng A.J., Sobolev A.P., Di Lorenzo A., Xiao J., Mannina L., Capitani D., Daglia M. (2019). Metabolite Characterization of Powdered Fruits and Leaves from *Adansonia digitata* L. (Baobab): A Multi-Methodological Approach. Food Chem..

[46] Coates J. (2006). Interpretation of Infrared Spectra, A Practical Approach. Encyclopedia of Analytical Chemistry.

[47] Kabra A., Sharma R., Hano C., Kabra R., Martins N., Baghel U.S. (2019). Phytochemical Composition, Antioxidant, and Antimicrobial Attributes of Different Solvent Extracts from *Myrica esculenta* Buch.-Ham. Ex. D. Don Leaves. Biomolecules.

[48] Thummajitsakul S., Samaikam S., Tacha S., Silprasit K. (2020). Study on FTIR Spectroscopy, Total Phenolic Content, Antioxidant Activity and Anti-Amylase Activity of Extracts and Different Tea Forms of *Garcinia schomburgkiana* Leaves. LWT.

[49] Etoundi C.B., Kuaté D., Ngondi J.L., Oben J. (2010). Anti-Amylase, Anti-Lipase and Antioxidant Effects of Aqueous Extracts of Some Cameroonian Spices. J. Nat. Prod..

[50] Peng X., Ma J., Chen F., Wang M. (2011). Naturally Occurring Inhibitors against the Formation of Advanced Glycation End-Products. Food Funct..

[51] Prakash O., Baskaran R., Kudachikar V.B. (2019). Characterization, Quantification of Free, Esterified and Bound Phenolics in Kainth (*Pyrus pashia* Buch.-Ham. Ex D.Don) Fruit Pulp by UPLC-ESI-HRMS/MS and Evaluation of Their Antioxidant Activity. Food Chem..

[52] Kuate D., Etoundi B.C.O., Soukontoua Y., Ngondi J., Oben J. (2011). Comparative Study of the Antioxidant, Free Radical Scavenging Activity and Human LDL Oxidation Inhibition of Three Extracts from Seeds of a Cameroonian Spice, *Xylopia parviflora* (A. Rich.) Benth (Annonaceae). Int. J. Biomed. Pharm. Sci..

[53] Manga E., Brostaux Y., Ngondi J.L., Sindic M. (2020). Optimisation of Phenolic Compounds and Antioxidant Activity Extraction Conditions of a Roasted Mix of *Tetrapleura tetraptera* (Schumach & Thonn.) and *Aframomum citratum* (*C. pereira*) Fruits Using Response Surface Methodology (RSM). Saudi J. Biol. Sci..

[54] Sokamte T.A., Mbougueng P.D., Tatsadjieu N.L., Sachindra N.M. (2019). Phenolic Compounds Characterization and Antioxidant Activities of Selected Spices from Cameroon. S. Afr. J. Bot..

[55] Daga P., Vaishnav S.R., Dalmia A., Tumaney A.W. (2021). Extraction, Fatty Acid Profile, Phytochemical Composition and Antioxidant Activities of Fixed Oils from Spices Belonging to Apiaceae and Lamiaceae Family. J. Food Sci. Technol..

[56] Daga P., Dalmia A., Vaishnav S.R., Tumaney A.W. (2022). Lipidome Analysis and Metabolite Profiling of Fixed Oils from Selected Spices. LWT.

[57] Mareček V., Mikyška A., Hampel D., Čejka P., Neuwirthová J., Malachová A., Cerkal R. (2017). ABTS and DPPH Methods as a Tool for Studying Antioxidant Capacity of Spring Barley and Malt. J. Cereal Sci..

[58] Singh Y., Datey A., Chakravortty D., Tumaney A.W. (2021). Novel Cell-Based Assay to Investigate Monoacylglycerol Acyltransferase 2 Inhibitory Activity Using HIEC-6 Cell Line. ACS Omega.

[59] Kim D.-O., Lee K.W., Lee H.J., Lee C.Y. (2002). Vitamin C Equivalent Antioxidant Capacity (VCEAC) of Phenolic Phytochemicals. J. Agric. Food Chem..

[60] Contreras T.C., Ricciardi E., Cremonini E., Oteiza P.I. (2015). (−)-Epicatechin in the Prevention of Tumor Necrosis Alpha-Induced Loss of Caco-2 Cell Barrier Integrity. Arch. Biochem. Biophys..

[61] Luo K.-W., Chen W., Lung W.-Y., Wei X.-Y., Cheng B.-H., Cai Z.-M., Huang W.-R. (2017). EGCG Inhibited Bladder Cancer SW780 Cell Proliferation and Migration Both in Vitro and in Vivo via Down-Regulation of NF-ΚB and MMP-9. J. Nutr. Biochem..

[62] Nani A., Murtaza B., Sayed Khan A., Khan N.A., Hichami A. (2021). Antioxidant and Anti-Inflammatory Potential of Polyphenols Contained in Mediterranean Diet in Obesity: Molecular Mechanisms. Molecules.

[63] Ruifeng G., Yunhe F., Zhengkai W., Ershun Z., Yimeng L., Minjun Y., Xiaojing S., Zhengtao Y., Naisheng Z. (2014). Chlorogenic Acid Attenuates Lipopolysaccharide-Induced Mice Mastitis by Suppressing TLR4-Mediated NF-ΚB Signaling Pathway. Eur. J. Pharmacol..

[64] Gonzales A.M., Orlando R.A. (2008). Curcumin and Resveratrol Inhibit Nuclear Factor-KappaB-Mediated Cytokine Expression in Adipocytes. Nutr. Metab..

[65] Zhang S., Xu M., Zhang W., Liu C., Chen S. (2021). Natural Polyphenols in Metabolic Syndrome: Protective Mechanisms and Clinical Applications. Int. J. Mol. Sci..

[66] Sharma S.A., Tummuru M.K.R., Blaser M.J., Kerr L.D. (1998). Activation of IL-8 Gene Expression by Helicobacter Pylori Is Regulated by Transcription Factor Nuclear Factor-ΚB in Gastric Epithelial Cells. J. Immunol..

[67] Yasumoto K., Okamoto S., Mukaida N., Murakami S., Mai M., Matsushima K. (1992). Tumor Necrosis Factor Alpha and Interferon Gamma Synergistically Induce Interleukin 8 Production in a Human Gastric Cancer Cell Line through Acting Concurrently on AP-1 and NF-KB-like Binding Sites of the Interleukin 8 Gene. J. Biol. Chem..

[68] Chen S.-J., Chung Y.-C., Chang H.-L., Chang H.-P., Chou J.-L., Lin C.-C., Chen C.-H., Hsu C.-P. (2020). Synergistic Effect of Combined Treatment with Longan Flower Extract and 5-Fluorouracil on Colorectal Cancer Cells. Nutr. Cancer.

[69] Pezzani R., Salehi B., Vitalini S., Iriti M., Zuñiga F.A., Sharifi-Rad J., Martorell M., Martins N. (2019). Synergistic Effects of Plant Derivatives and Conventional Chemotherapeutic Agents: An Update on the Cancer Perspective. Medicina.

[70] Chamberlin S.R., Blucher A., Wu G., Shinto L., Choonoo G., Kulesz-Martin M., McWeeney S. (2019). Natural Product Target Network Reveals Potential for Cancer Combination Therapies. Front. Pharmacol..

