# Supplementary material for: Anti-Inflammatory, Antioxidant Activities, and Phytochemical Characterization of Edible Plants Exerting Synergistic Effects in Human Gastric Epithelial Cells
Source: Antioxidants (Basel). 2023 Feb 27;12(3):591. doi: 10.3390/antiox12030591 (PMC10045632; doi:10.3390/antiox12030591)
Supplement: Supplementary file 1 [file antioxidants-12-00591-s001.zip › antioxidants-2180393-supplementary.pdf]

Figure S1. UPLC-ESI-HRMS/MS mass spectra of *Aframomum citratum (C.Pereira) K.Schum* **(A)**, *Dichrostachys glomerata (Forssk.) Chiov.* **(B)**, *Tetrapleura tetraptera (Schum. and Thonn.)Taub* **(C)** and *Xylopia parviflora Spruce* **(D)** extracts.

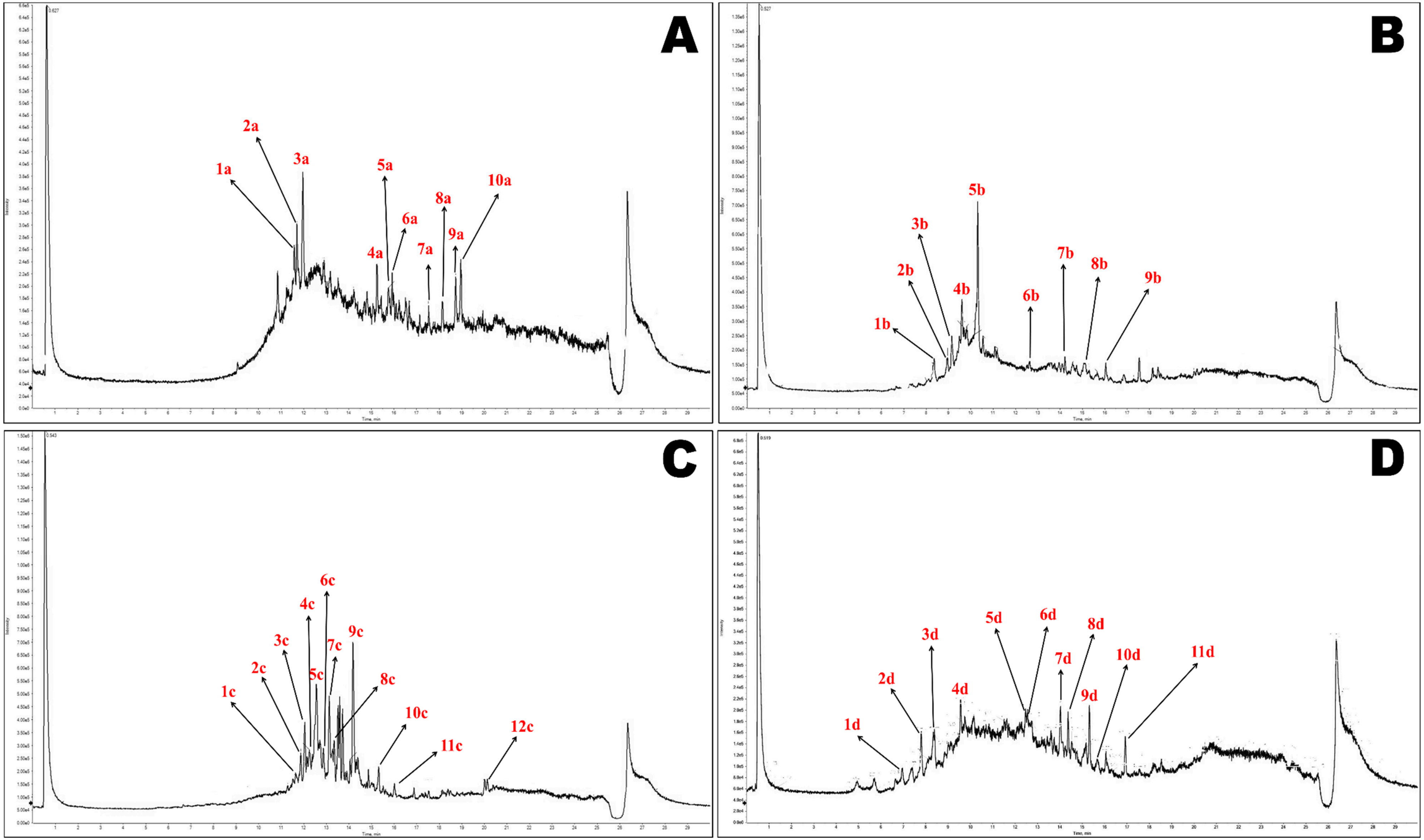

Figure S2. FTIR spectra of *Aframomum citratum (C.Pereira) K.Schum* **(A)**, *Dichrostachys glomerata (Forssk.) Chiov.* **(B)**, *Tetrapleura tetraptera (Schum. and Thonn.)Taub* **(C)** and *Xylopia parviflora Spruce* **(D)** extracts.

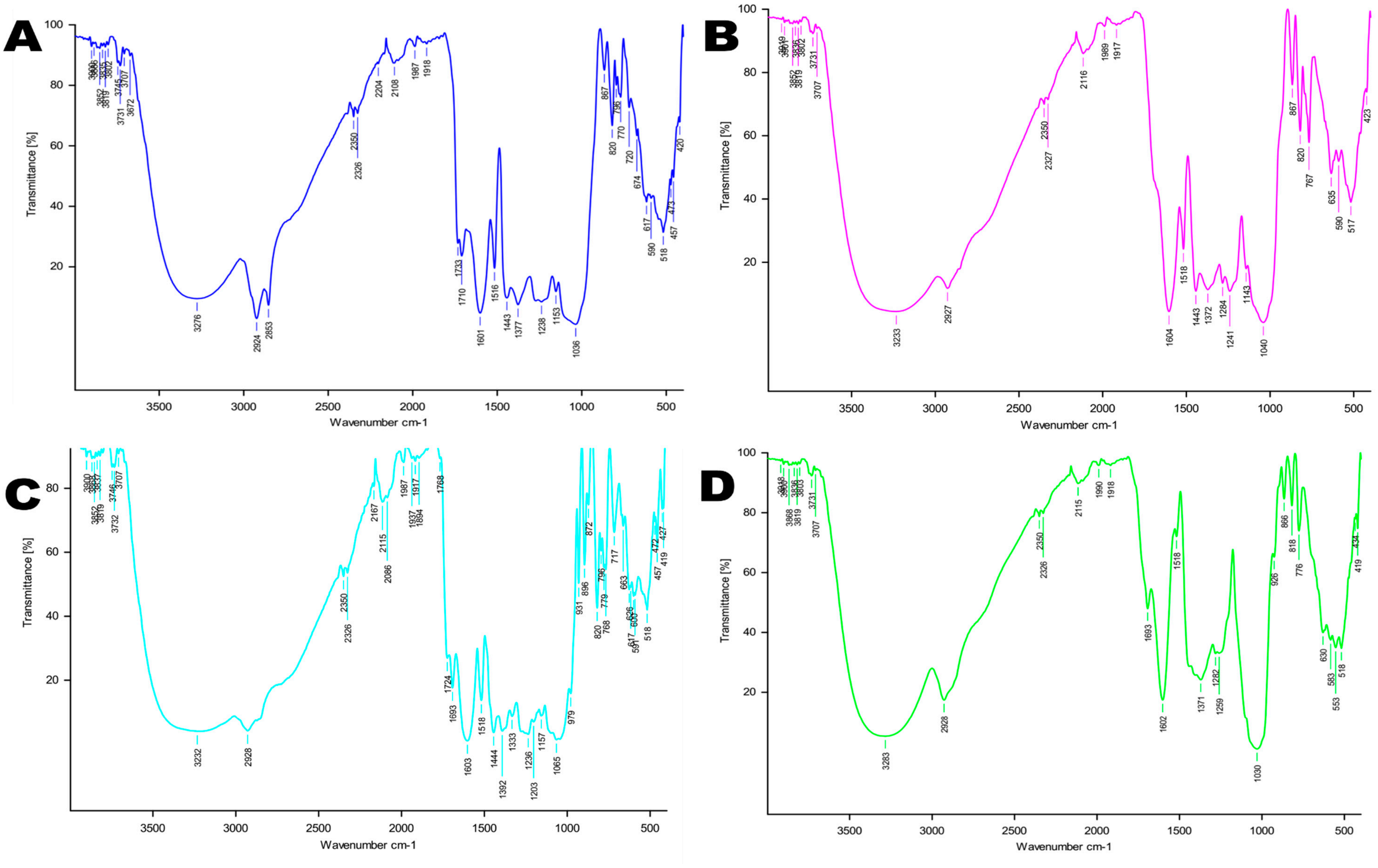

Table S1. Correlation coefficient (R) among the assays (antioxidant assays with phenolic compounds)

Variables	TPC	TFC	FC	ABTS	DPPH	Protocatecheuic acid	Epicatechin	Catechin	Caffeic acid	p-Coumaric acid	Rosmarinic acid	Kaempferol
TPC	1											
TFC	**0.99***	1										
FC	0.30	0.41	1									
ABTS	**0.97***	**0.99***	-0.54	1								
DPPH	**0.97***	**0.96***	-0.44	**0.97***	1							
Protocatecheuic acid	-0.66	-0.68	0.06	0.58	0.46	1						
Epicatechin	0.52	0.41	-0.59	-0.29	-0.45	-0.33	1					
Catechin	0.52	0.41	-0.59	-0.29	-0.45	-0.33	**1***	1				
Caffeic acid	0.52	0.41	-0.59	-0.29	-0.45	-0.33	**1***	**1***	1			
p-Coumaric acid	0.39	0.48	**0.99***	-0.61	-0.54	0.05	-0.49	-0.49	-0.49	1		
Rosmarinic acid	0.51	0.60	**0.97***	-0.71	-0.62	-0.13	-0.42	-0.42	-0.42	**0.98***	1	
Kaempferol	-0.75	-0.76	0.01	0.67	0.57	**0.99***	-0.39	-0.39	-0.39	-0.01	-0.18	1

(*): Significant ($p<0.05$) between variables. **ABTS:** 2,2'-azinobis-(3-ethylbenzothiazoline-6-sulfonic acid); **DPPH:** 1,1-Diphenyl-2-picryl-hydrazyl; **FC:** Flavonol content; **TPC:** Total phenol content; **TFC:** Total flavonoid content.

Figure S3. Principal component analysis (PCA) of the antioxidant assays, phenolic acids and flavonoids determined by HPLC-PDA

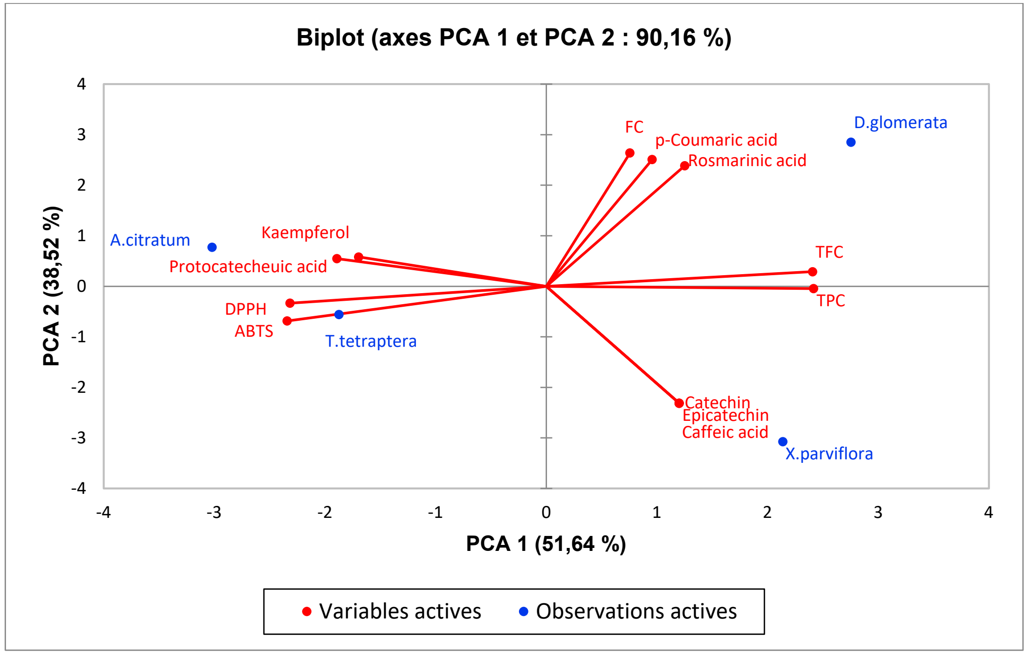

Figure S4. Microscopy (inverted phase contrast microscopy; 10X magnification) representation of human normal gastric (GES-1) cells **(A)** gastric adenocarcinoma (AGS) **(B)** epithelial cells.

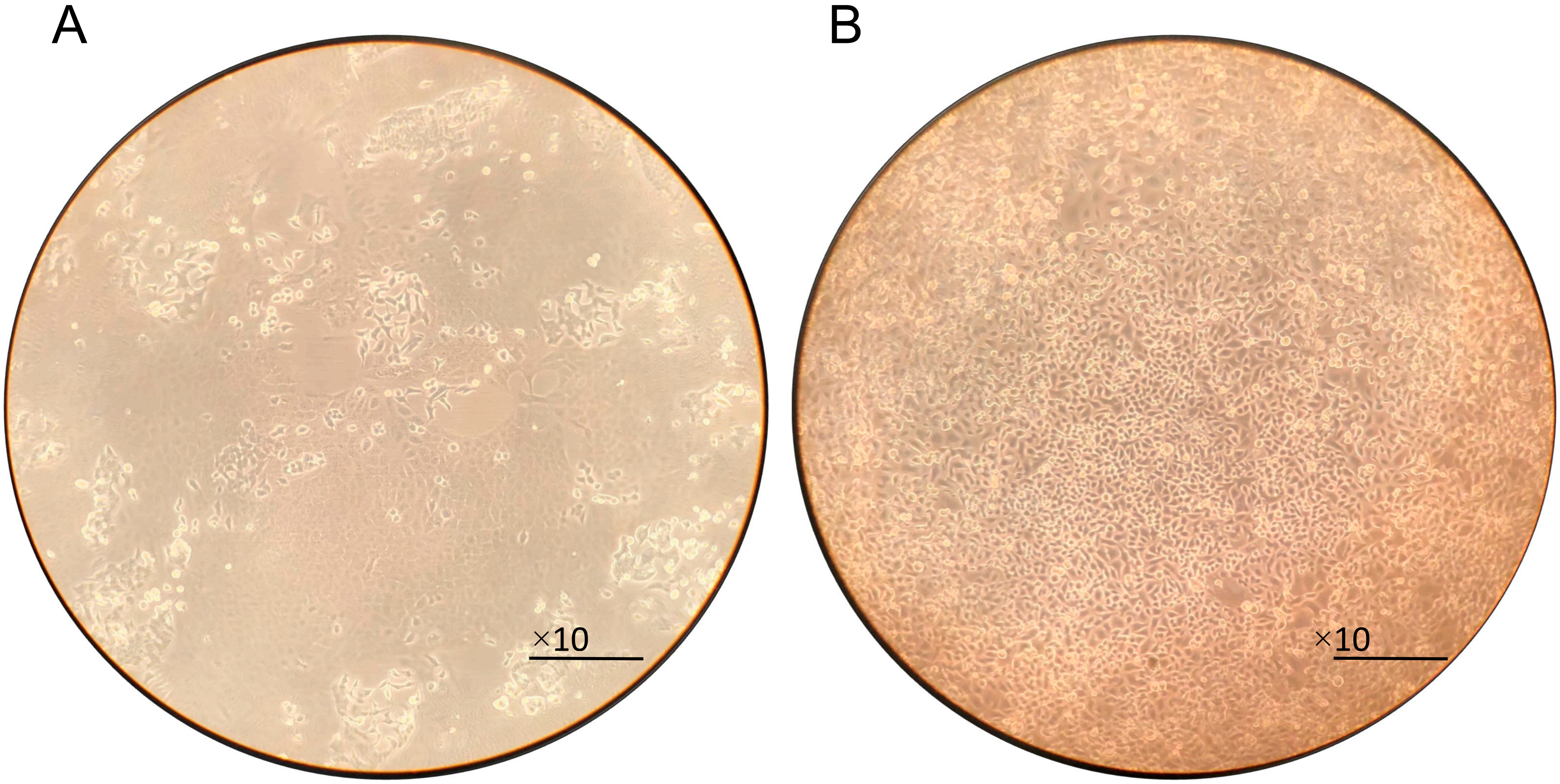

Figure S5. Effect of extracts in monotherapy and combination on the basal level of NF-κB-driven transcription in non-stimulated human gastric adenocarcinoma (AGS) and gastric epithelial (GES-1) cells.

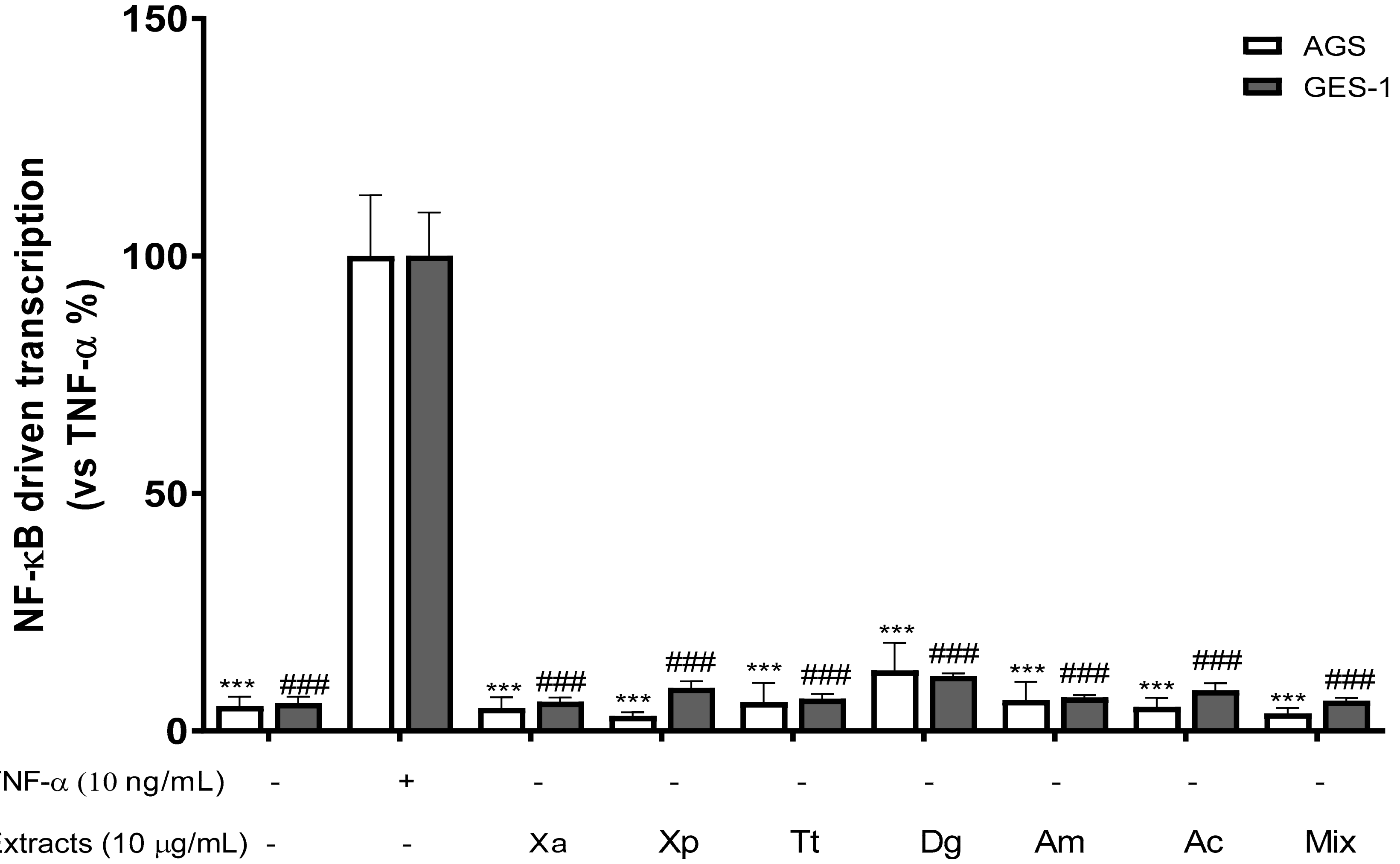

Data are expressed as percentages versus the stimulated control, which is arbitrarily set to 100%. * $p < 0.05$; ** $p < 0.01$; *** $p < 0.001$ in AGS cells and $^{\#}p < 0.05$, $^{\#\#}p < 0.01$ and $^{\#\#\#}p < 0.001$ in GES-1 cells.
